# Intermittent supplementation with fisetin improves arterial function in old mice by decreasing cellular senescence

**DOI:** 10.1111/acel.14060

**Published:** 2023-12-07

**Authors:** Sophia A. Mahoney, Ravinandan Venkatasubramanian, Mary A. Darrah, Katelyn R. Ludwig, Nicholas S. VanDongen, Nathan T. Greenberg, Abigail G. Longtine, David A. Hutton, Vienna E. Brunt, Judith Campisi, Simon Melov, Douglas R. Seals, Matthew J. Rossman, Zachary S. Clayton

**Affiliations:** ^1^ Department of Integrative Physiology University of Colorado Boulder Boulder Colorado USA; ^2^ Buck Institute for Research on Aging Novato California USA; ^3^ Lawrence Berkeley National Laboratory Berkeley California USA

**Keywords:** aging, arterial stiffness, cellular senescence, endothelial function, nutraceutical, vascular dysfunction

## Abstract

Cellular senescence and the senescence‐associated secretory phenotype (SASP) contribute to age‐related arterial dysfunction, in part, by promoting oxidative stress and inflammation, which reduce the bioavailability of the vasodilatory molecule nitric oxide (NO). In the present study, we assessed the efficacy of fisetin, a natural compound, as a senolytic to reduce vascular cell senescence and SASP factors and improve arterial function in old mice. We found that fisetin decreased cellular senescence in human endothelial cell culture. In old mice, vascular cell senescence and SASP‐related inflammation were lower 1 week after the final dose of oral intermittent (1 week on—2 weeks off—1 weeks on dosing) fisetin supplementation. Old fisetin‐supplemented mice had higher endothelial function. Leveraging old p16‐3MR mice, a transgenic model allowing genetic clearance of p16^INK4A^‐positive senescent cells, we found that ex vivo removal of senescent cells from arteries isolated from vehicle‐ but not fisetin‐treated mice increased endothelium‐dependent dilation, demonstrating that fisetin improved endothelial function through senolysis. Enhanced endothelial function with fisetin was mediated by increased NO bioavailability and reduced cellular‐ and mitochondrial‐related oxidative stress. Arterial stiffness was lower in fisetin‐treated mice. Ex vivo genetic senolysis in aorta rings from p16‐3MR mice did not further reduce mechanical wall stiffness in fisetin‐treated mice, demonstrating lower arterial stiffness after fisetin was due to senolysis. Lower arterial stiffness with fisetin was accompanied by favorable arterial wall remodeling. The findings from this study identify fisetin as promising therapy for clinical translation to target excess cell senescence to treat age‐related arterial dysfunction.

## Graphical abstract

Oral intermittent fisetin supplementation reduces vascular cell senescence to improve vascular endothelial function and reduce aortic stiffness in old mice.

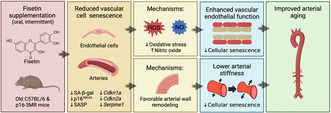

**Abbreviations**
ACh: Acetylcholine
AGEs: Advanced glycation end products
CuZn: Copper zinc
CVD: Cardiovascular diseases
EDD: Endothelium dependent dilation
GCV: Ganciclovir
HAECs: Human aortic endothelial cells
HUVECs: Human umbilical vein endothelial cells
L‐NAME: N(gamma)‐nitro‐L‐arginine methyl ester
Mn: Manganese
NADPH: Nicotinamide adenine dinucleotide phosphate
NO: Nitric oxide
PWV: Pulse wave velocity
ROS: Reactive oxygen species
SA‐β‐gal: Senescence‐associated beta galactosidase
SASP: senescence‐associated secretory phenotype
SNP: Sodium nitroprusside
SOD: Superoxide dismutase
TEMPOL: 4‐hydroxy‐2,2,6,6‐tetramethylpiperidin‐1‐oxyl

## 1 INTRODUCTION

Advancing age is the primary risk factor for cardiovascular diseases (CVDs), which remain the leading cause of morbidity and mortality worldwide (Benjamin et al., 2018). Arterial dysfunction, characterized by vascular endothelial dysfunction and large elastic artery stiffening (Jia et al., 2019), is a key antecedent to the development of clinical CVDs with aging.

Age‐related declines in endothelial function, as shown by reduced endothelium‐dependent dilation (EDD), are mediated primarily by lower bioavailability of nitric oxide (NO) (Brunt et al., 2019; El Assar et al., 2012; Lakatta & Levy, 2003; Seals et al., 2011). Age‐associated arterial stiffening, as indicated by an increase in aortic pulse wave velocity (PWV), is largely due to increased aortic intrinsic mechanical wall stiffness (Laurent et al., 2003; Vlachopoulos et al., 2010) and arterial wall remodeling, featuring collagen deposition (fibrosis), elastin degradation and crosslinking of these structural proteins by advanced glycation end products (AGEs) (Clayton, Brunt, et al., 2021; Zieman et al., 2005). Reduced NO‐mediated endothelial function and arterial stiffening are driven by excess reactive oxygen species (ROS)‐related oxidative stress, a key source being dysfunctional mitochondria (Rossman et al., 2020), and chronic low‐grade inflammation (Steven et al., 2019). Thus, identifying therapies that reduce oxidative stress and inflammation to improve arterial function with aging is an important biomedical research goal (Fleenor et al., 2012, 2013; Lesniewski et al., 2011).

Cellular senescence is a cellular stress response characterized by essentially irreversible cell cycle arrest (Campisi & d'Adda di Fagagna, 2007; Kuehnemann et al., 2023). Homeostatic levels of cellular senescence aids in wound healing and inhibits tumorigenesis in part through the production of the senescence‐associated secretory phenotype (SASP)—a collection of cytokines, chemokines, and inflammatory mediators—which activate immune responses (Campisi, 2013; Campisi & d'Adda di Fagagna, 2007; Demaria et al., 2014). Senescent cells accumulate in excess with aging leading to adverse cellular effects in part via the SASP, which contributes to a pro‐inflammatory and pro‐oxidative environment (Campisi & d'Adda di Fagagna, 2007; van Deursen, 2014). Cellular senescence is a mechanism of age‐related arterial dysfunction (Clayton et al., 2023; Roos et al., 2016) and increased cellular senescence in vascular cells from older adults is associated with impaired arterial function (Rossman et al., 2017). Thus, reducing cellular senescence and the SASP in the vasculature may be a promising therapeutic approach for improving arterial function in old age.

Clinical trials of senolytics—compounds that clear senescent cells—are underway, but the impact and safety of current synthetic senolytic therapies being tested are not fully understood (Hickson et al., 2019; Justice et al., 2019). Fisetin is a flavonoid found in a variety of commonly consumed foods, appears to be safe in humans and has high translational potential (Bondonno et al., 2019; Farsad‐Naeimi et al., 2018; Hejazi et al., 2020; Khan et al., 2013; Kim & Je, 2017; Wang et al., 2019; Yousefzadeh et al., 2018). Intermittent fisetin treatment—to clear excess senescent cells, but not interfere with the homeostatic functions of cellular senescence (i.e., wound healing)—reduces cellular senescence in select tissues and extends life span without adverse effects in old mice (Yousefzadeh et al., 2018). However, previous studies have limited physiological outcomes and it is unknown if intermittent fisetin supplementation reduces vascular cell senescence and improves arterial function with aging.

The purpose of this study was to assess the efficacy of fisetin for reducing cellular senescence and the SASP in vascular cells and arteries and improving arterial function in old mice. We first sought to identify concentrations of fisetin that selectively reduce cellular senescence in endothelial cells in culture. Using a dose of fisetin based on our findings in cell culture, we set out to determine if oral, intermittent fisetin treatment reduced vascular senescent cell burden and SASP‐related inflammation in old mice. We then assessed if fisetin could improve endothelial function and arterial stiffness by decreasing cellular senescence. Lastly, we determined if fisetin improved endothelial function by increasing NO bioavailability and reducing total and mitochondrial ROS, and if reductions in arterial stiffness were accompanied by favorable arterial wall remodeling.

## 2 METHODS

### 2.1 Cell culture experiments.

HUVECs and HAECs (Lonza, Basel, Switzerland) were cultured at 37°C and 5% CO_2_ to ~80% confluency in Endothelial Cell Growth Media (EGM)‐2 media (Lonza) supplemented with an additional 2% fetal calf serum (FCS; Sigma‐Aldrich Corp., St. Louis, MO), 100 μg/mL penicillin, and 172 μg/mL streptomycin (Gibco, Gaithersburg, MD). Nonsenescent (control) cells were passaged 3–6 times, and senescent cells were grown to passage 15 to induce replicative senescence, as previously shown (Hayflick & Moorhead, 1961). Senescent cells were treated with fisetin in EGM‐2 for 48 h, and cell assays were immediately performed in triplicate following the treatment period (Udvardi et al., 2008). Experimental details on the cell viability assay, SA‐β‐gal staining, assessment of ROS levels, and gene expression are provided in the Appendix S1.

### 2.2 Ethical approval and animal studies

All mouse studies and procedures were reviewed and approved by the Institutional Animal Care and Use Committee at the University of Colorado Boulder (Protocol No. 2618). All procedures adhered to the guidelines set forth by the National Institutes of Health's Guide for the Care and Use of Laboratory Animals (Animals, 2011).

Male C57BL/6N (wild type) mice were obtained from the National Institute of Aging colony (maintained by Charles River, Wilmington, MA). Wild‐type mice were allowed to acclimate to our facilities for 4 weeks before beginning the study. Male and female p16‐3MR mice were bred and aged in our mouse colony at the University of Colorado Boulder. These mice carry a trimodal fusion protein (3MR) under the control of the p16^INK4A^ promoter which allows for selective genetic clearance of excess p16^INK4A^‐positive senescent cells by administering the antiviral agent GCV (Demaria et al., 2014). For the duration of the study, all mice were single housed at our animal facility with a 12 h:12 h light–dark cycle and allowed ad libitum access to an irradiated, fixed, and open rodent chow (Inotiv/Envigo 7917, stored at room temperature) and drinking water.

For the intervention period, old (27 months) wild‐type and p16‐3MR mice were assigned to receive vehicle (10% EtOH, 30% PEG400, and 60% Phosal 50 PG) or fisetin (100 mg/kg/day in vehicle). For wild‐type mice, 18 mice received vehicle and 19 mice received fisetin, and for p16‐3MR mice, 27 mice received vehicle and 35 mice received fisetin. Treatment was administered via oral gavage using an intermittent dosing paradigm involving 1 week of daily active dosing, 2 weeks of no intervention, and then another 1 week of active dosing, which has previously been shown to reduce senescent cell burden in select tissues in old mice back to young/basal levels (Yousefzadeh et al., 2018). Mice were sacrificed 1–2 weeks after the final dose. Treatment groups were randomized and matched for baseline body weight and aortic PWV. Throughout the intervention period, one old wild‐type mouse and 11 p16‐3MR mice died as a result of expected age‐related attrition, which resulted in a final sample size of old wild‐type vehicle, *n* = 17; old wild‐type fisetin, *n* = 19; old p16‐3MR vehicle, *n* = 22; and old p16‐3MR fisetin, *n* = 29.

### 2.3 In vivo aortic stiffness and arterial blood pressure

Aortic PWV was assessed in vivo 1 week before (pre) and 1 week after (post) the intervention, as previously described (Brunt et al., 2019; Casso et al., 2022; Clayton, Brunt, et al., 2021; Clayton, Hutton, et al., 2021). Briefly, mice were anesthetized via inhaled isoflurane (1.0%–2.5%) and positioned supine on a warmed heat platform with paws secured to electrocardiogram (ECG) leads. Two Doppler probes were then placed at the transverse aortic arch and the abdominal aorta. Three repeated 2‐second ultrasound tracings were recorded, and average pre‐ejection time (i.e., time between the R‐wave of the ECG to the foot of the Doppler signal) was determined for each location. Aortic PWV was then calculated as: aortic PWV = (physical distance between the two probes)/(∆time_abdominal_ minus ∆time_transverse_) and reported in centimeters per second. Blood pressure was measured pre‐ and post‐intervention on 3 consecutive days using a noninvasive tail‐cuff method (CODA; Kent Scientific, Torrington, CT), as we have described previously (Brunt et al., 2019; Casso et al., 2022; Clayton, Brunt, et al., 2021; Clayton, Hutton, et al., 2021).

### 2.4 Endothelial function

EDD in response to increasing doses of acetylcholine (Sigma Aldrich, Cat. No. A6625) and endothelium‐independent dilation in response to increasing concentrations of the exogenous NO donor SNP (Sigma Aldrich, Cat. No. 13755–38‐9) were measured in isolated carotid arteries, as previously described (Brunt et al., 2019; Casso et al., 2022; Clayton et al., 2020; Clayton, Hutton, et al., 2021). Further details, including all pharmacological agents and approaches used for pharmacodissection experiments, are provided in the Appendix S1.

### 2.5 Statistical analyses

Detailed descriptions of all statistical analyses performed are provided in the Appendix S1. Data are presented as mean ± SEM in text, figures, and tables unless specified otherwise. Statistical significance was set to α = 0.05. All statistical analyses were performed using Prism, version 9 (GraphPad Software, Inc, La Jolla, CA).

## 3 RESULTS

### 3.1 Fisetin reduces cellular senescence in endothelial cells

**FIGURE 1:**
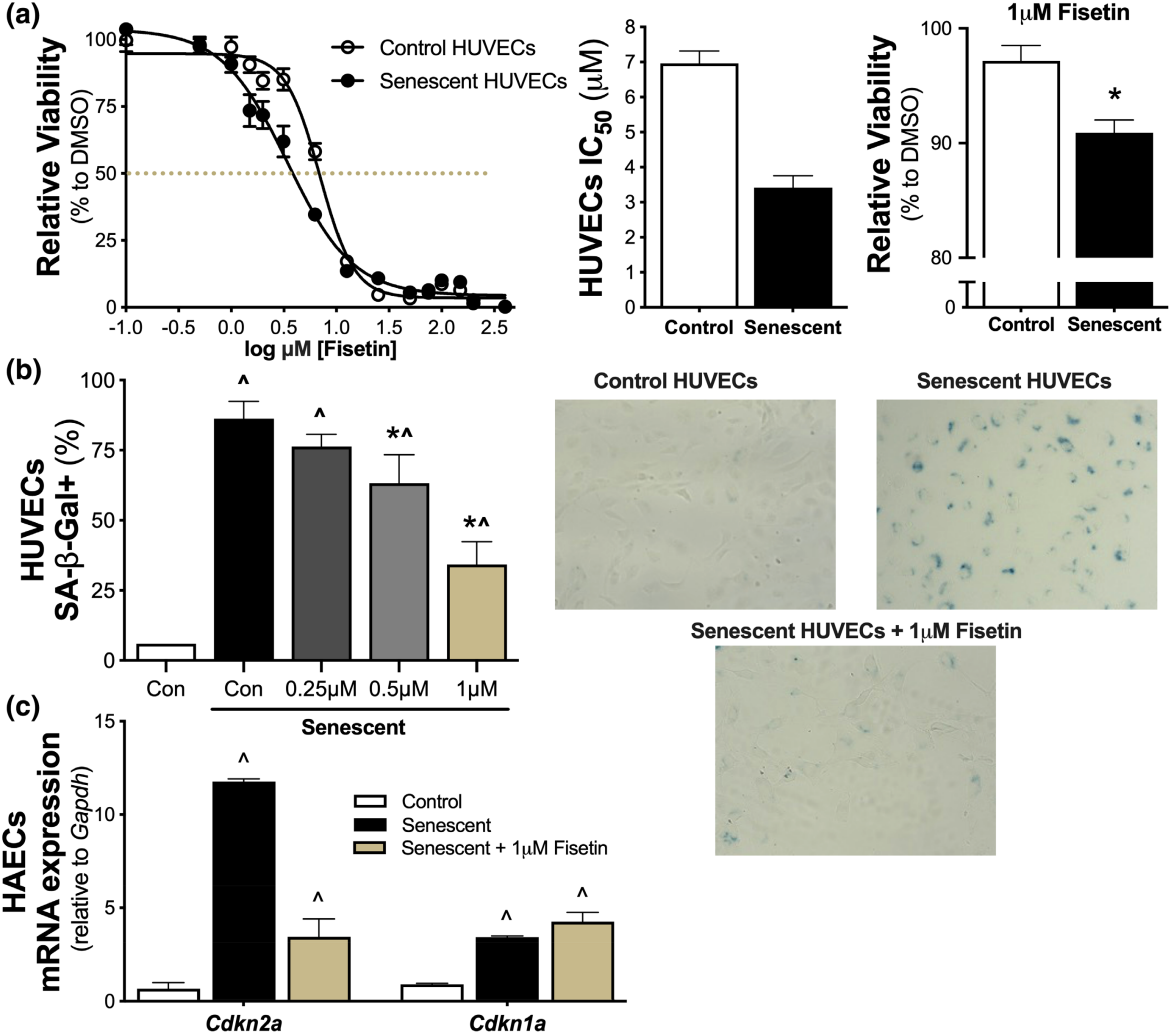
Fisetin suppresses cellular senescence in endothelial cells. Cell viability in replicating (control) and senescent human umbilical vein endothelial cells (HUVECs) to increasing doses of fisetin (dashed line represents the IC_50_). IC_50_ values of control and senescent HUVECs with 95% confidence intervals. Viability of control and senescent HUVECs at 1 μM fisetin (*n* = 4–8) (a). Senescence‐associate beta galactosidase (SA‐β‐Gal) signal in control and senescent HUVECs to increasing doses of fisetin (*n* = 3) and representative images, right (b). mRNA gene expression of cellular senescence markers *Cdkn2a* and *Cdkn1a* in control, senescent, or 1 μM fisetin‐treated senescent human aortic endothelial cells (HAECs) (*n* = 3) (c). Values represent mean ± SEM. *p < 0.05 senescent versus senescent + treatment; ^*p* < 0.05 versus control.

Initially, we performed in vitro cell culture experiments in human umbilical vein endothelial cells (HUVECs) to determine the effect of fisetin on senescent and nonsenescent (control) endothelial cells. HUVECs were brought to senescence via replicative exhaustion (an in vitro model of an aged‐like state) (Cristofalo et al., 2004; Hayflick & Moorhead, 1961). Cell viability of senescent and control HUVECs were measured in response to increasing concentrations of fisetin to determine the concentrations of fisetin that reduced the viability of senescent cells without affecting viability of control cells. We found that fisetin had an overall higher potency against senescent HUVECs compared to control cells (IC_50_ of fisetin 7.0 ± 0.4 vs. 3.4 ± 0.3 μM on control and senescent cells, respectively; Figure 1a). Moreover, we observed a reduction in viability of senescent HUVECs with 1 μM fisetin, whereas no significant effects on viability were observed on control HUVECs at this concentration (viability at 1 μM fisetin: control cells, 97 ± 1% vs. senescent cells, 91 ± 1%, *p* = 0.003; Figure 1a). Based on these observations, we selected 1 μM as the maximal concentration of fisetin for the remainder of the cell culture experiments, as this concentration appeared to selectively influence senescent cell viability without affecting nonsenescent cells.

To determine if the decrease in viability of senescent cells with fisetin treatment was associated with reductions in cellular senescence, we assessed the senescence‐associated β‐galactosidase (SA‐β‐gal) signal—an established hallmark of cellular senescence (Kurz et al., 2000)—in control and senescent HUVECs exposed to increasing concentrations of fisetin up to 1 μM. We found that senescent HUVECs had 13.5‐fold higher SA‐β‐gal signal relative to nonsenescent control HUVECs (*p* = 0.002) and that fisetin reduced the SA‐β‐gal signal in a concentration‐dependent manner up to 1 μM (Figure 1b and Figure S1).

After establishing the effect of fisetin in senescent HUVECs, we aimed to extend our understanding of the senolytic effects of fisetin in arterial cells using human aortic endothelial cells (HAECs). Accordingly, in HAECs, we measured gene expression of *Cdkn2a* and *Cdkn1a*, which encode the cyclin‐dependent kinase inhibitor proteins p16^INK4A^ and p21^Cip1/Waf1^, respectively. Compared to control cells, these cyclin‐dependent kinase inhibitors are observed at increased levels in most senescent cells and are largely responsible for the irreversible cell cycle arrest associated with cellular senescence (Demaria et al., 2014; González‐Gualda et al., 2021). Senescent HAECs demonstrated 17‐fold (*p* < 0.0001) and 4‐fold (*p* < 0.0001) higher expression of *Cdkn2a* and *Cdkn1a*, respectively, compared to control HAECs (Figure 1c). In replicative senescent HAECs, 1 μM fisetin lowered the expression of *Cdkn2a* by 71% (*p* < 0.0001) back toward levels of control cells but had no effect on the relatively modest increase in *Cdkn1a* expression, suggesting that fisetin may preferentially act upon the p16^INK4A^, rather than the p21^Cip1/Waf1^, transcription factor cascade to eliminate senescent endothelial cells (Figure 1c).

Collectively, we observed a reduction in viability of senescent endothelial cells treated with 1 μM fisetin, but no effects on viability of control cells treated with this concentration. In senescent endothelial cells treated with fisetin, we found decreases in select markers of cellular senescence. Thus, our in vitro experiments demonstrate that fisetin can selectively target excess cellular senescence in vascular endothelial cells.

### 3.2 Animal characteristics

**FIGURE 2:**
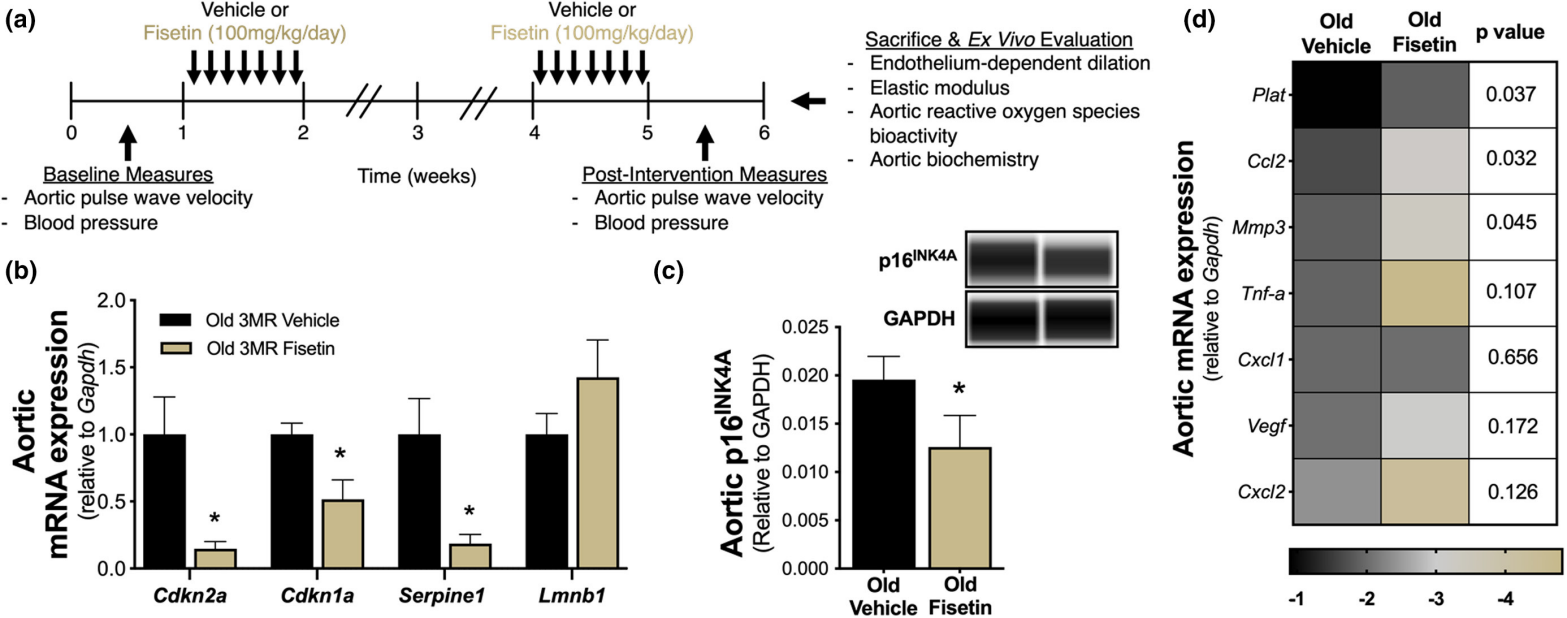
Fisetin reduces cellular senescence in the vasculature of old mice. Study design used in the animal model (a). Aortic mRNA gene expression of cellular senescence markers in old p16‐3MR (3MR) mice (*n* = 12–15) (b). Aortic protein abundance of p16^INK4A^ in wild‐type mice with representative virtual blot bands (*n* = 8) (c). Aortic mRNA gene expression of senescence‐associated secretory phenotypes factors (*n* = 14–15) (d). Color intensities represent log_2_‐fold changes. Values represent mean ± SEM. *p < 0.05 vehicle versus fisetin.

We next sought to extend the senolytic effects of fisetin to in vivo arterial aging by assessing cellular senescence in intact arteries and arterial function of aged mice intermittently supplemented with fisetin. For the intervention, old male wild‐type received vehicle (10% EtOH, 30% PEG400 and 60% Phosal 50 PG) or fisetin (100 mg/kg/day in the vehicle). After establishing the effects of fisetin in male wild‐type mice, we used the same intervention regimen to look at sex differences and dissect the role of cellular senescence in old male and female p16‐3MR mice (Demaria et al., 2014, 2017). Since we did not observe sex differences in any of the outcomes in p16‐3MR mice, male and female data were combined in the results. The dose of fisetin was chosen based on the in vivo translation of the 1 μM concentration used in our cell culture experiments, as peak plasma levels of fisetin reach ~1 μM following oral ingestion of fisetin at a dose of 100 mg/kg/day in mice (Jo et al., 2016; Touil et al., 2011). Treatment was administered via oral gavage using an intermittent dosing paradigm—1 week on; 2 weeks off; 1 week on, as previously described (Yousefzadeh et al., 2018) (Figure 2a). Functional and biochemical measurements were assessed 1 week after the final dose.

Blood pressure, total body mass, mass of key tissues, and carotid artery and aorta characteristics at time of euthanasia in the vehicle‐ and fisetin‐treated wild‐type mice are presented in Table 1 (p16‐3MR animal characteristics are presented in Table S1). Fisetin did not affect any of these characteristics in either mouse model (*p* > 0.05).

**TABLE 1:** Wild‐type animal characteristics.

Characteristics	Vehicle	Fisetin
*n*	15	19
Body mass, g	29.3 ± 0.5	28.0 ± 0.7
Heart mass, mg	152 ± 4	165 ± 6
Quadriceps mass, mg	271 ± 11	280 ± 11
Visceral adipose mass, mg	324 ± 31	292 ± 44
Liver mass, g	1.6 ± 0.1	1.4 ± 0.1
Spleen mass, mg	82.4 ± 7.6	82.7 ± 8.5
Carotid artery		
Resting diameter, μm	428 ± 15	412 ± 13
Maximal diameter, μm	526 ± 11	519 ± 9
Aorta		
Diameter, μm	416 ± 10	415 ± 8
Intima media thickness, μm	40 ± 2	39 ± 2
Systolic blood pressure, mmHg		
Pre	96 ± 2	94 ± 2
Post	94 ± 2	89 ± 2
Diastolic blood pressure, mmHg		
Pre	73 ± 2	73 ± 2
Post	75 ± 2	71 ± 2

*Note*: Values represent mean ± SEM.

### 3.3 Fisetin targets cellular senescence and the SASP in the vasculature of old mice

To establish the senolytic effects of fisetin in the vasculature of old mice, we assessed markers of cellular senescence in mouse aorta lysates. We found that aortic gene expression of the cellular senescence markers *Cdkn2a* (−85%, *p* = 0.010), *Cdkn1a* (−48%, *p* = 0.006), and *Serpine1* (−81%, *p* = 0.011) were lower in old fisetin‐supplemented p16‐3MR mice compared to vehicle‐treated p16‐3MR mice, whereas no statistical differences were observed in *Lmnb1* expression (+45%, *p* = 0.213), a contributor of chromatin stability known to be reduced in senescent cells (González‐Gualda et al., 2021) (Figure 2b). Given that we observed a general reduction in vascular cell senescence transcripts in our initial gene expression screening, we validated these differences by measuring vascular protein abundance of p16^INK4A^. We found that old wild‐type mice supplemented with fisetin had 36% lower aortic p16^INK4A^ protein abundance (*p =* 0.007; Figure 2c and Figure S2a) compared to old vehicle‐treated mice. Fisetin supplementation also lowered the aortic expression of several SASP factors, including pro‐inflammatory cytokines (*Tnfα*; *P =* 0.107), chemokines (*Ccl2*, *Cxcl2*; *p =* 0.032 and *p =* 0.126, respectively), growth factors (*Vegf*; *P =* 0.172), and proteinases (*Mmp3*, *Plat*; *p =* 0.045 and *p =* 0.037, respectively) (Figure 2d). These results suggest that intermittent fisetin supplementation can decrease cellular senescence and modulate SASP factors in the vasculature of old mice.

### 3.4 Fisetin improves endothelial function in old mice by suppressing cellular senescence, enhancing NO bioavailability, and abolishing oxidative stress and its associated suppression of EDD

#### 3.4.1 EDD

**FIGURE 3:**
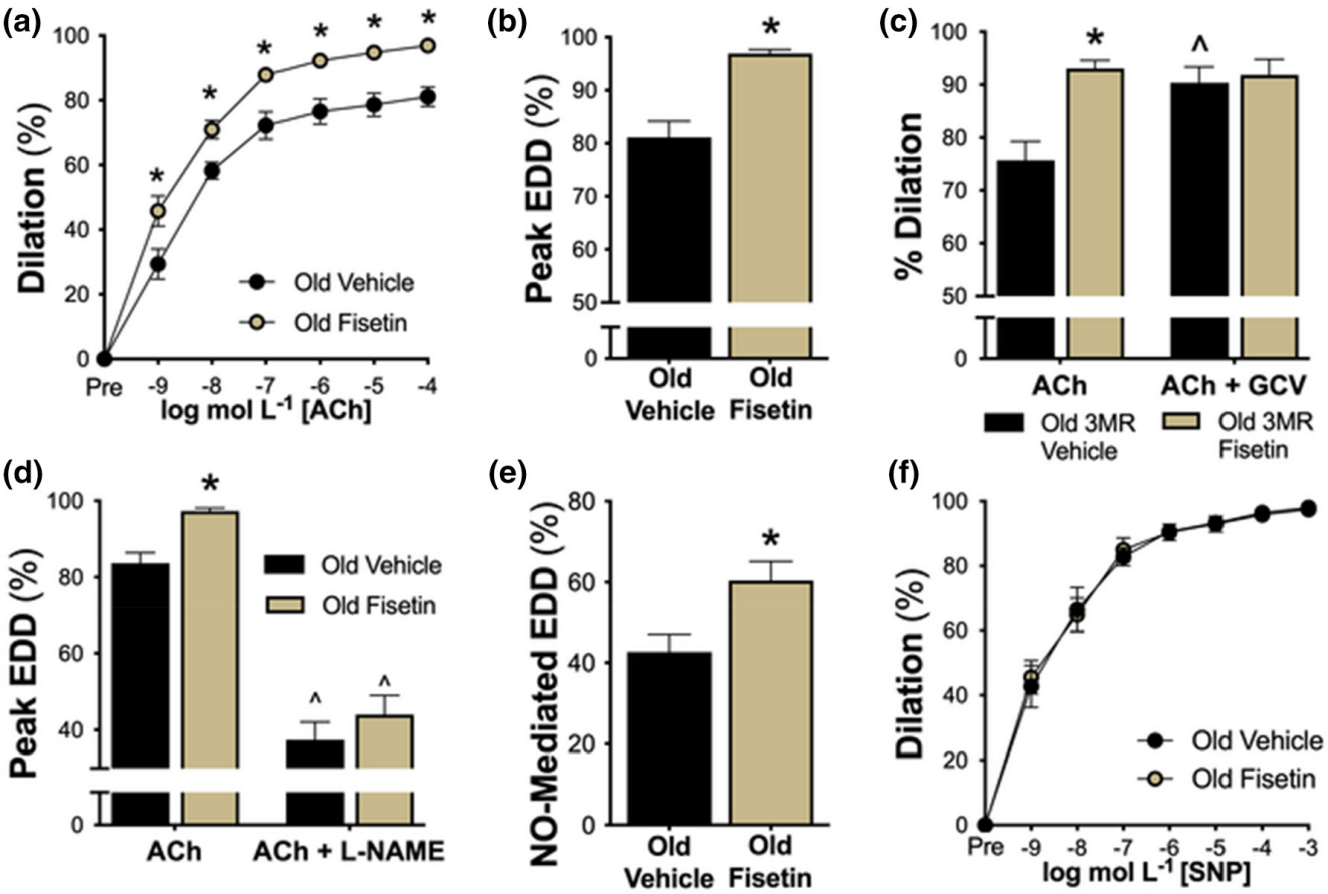
Fisetin improves endothelial function in old mice by suppressing cellular senescence and increasing nitric oxide (NO) bioavailability. Endothelium‐dependent dilation (EDD) in isolated carotid arteries in response to acetylcholine (ACh; 1 × 10^−9^ to 1 × 10^−4^ M; *n* = 12–18) (a,b). EDD in response to ACh in the presence or absence of ex vivo genetic elimination of cellular senescence via ganciclovir (GCV; 5 μm; 180 min preincubation; *n* = 9–11) (c). NO‐mediated EDD in response to ACh in the presence or absence of the NO‐synthase inhibitor, L‐NAME (0.1 mM; 30 min preincubation; *n* = 15) (d,e). Endothelium‐independent dilation to increasing doses of the NO donor, sodium nitroprusside (SNP; 1 × 10^−10^ to 1 × 10^−4^ M; *n* = 9) (f). Values represent mean ± SEM. *p < 0.05 old vehicle versus old fisetin; ^*p* < 0.05 versus ACh alone.

To determine whether fisetin improves endothelial function in advanced age, we assessed EDD ex vivo in carotid arteries excised from old wild‐type vehicle‐ and fisetin‐supplemented mice in response to increasing doses of acetylcholine (ACh). EDD was 20% greater in old mice supplemented with fisetin relative to old vehicle‐treated mice (peak EDD: vehicle, 81 ± 3% vs. fisetin, 97 ± 1%, *p* < 0.001; Figure 3a,b), suggesting that intermittent fisetin supplementation improves vascular endothelial function in old age.

#### 3.4.2 Cellular senescence‐mediated suppression of EDD

Following initial EDD measurements, we aimed to acquire causal evidence that reductions in cellular senescence with fisetin mediate improvements in endothelial function using “pharmacodissection” techniques. To accomplish this, we utilized the p16‐3MR transgenic mouse model which allows for genetic clearance of p16^INK4A^‐positive senescent cells with GCV in vivo and ex vivo (Demaria et al., 2014). Leveraging genetic senolysis models in combination with senolytic agents is considered the reference standard for assessing the causal impact of cellular senescence on physiological function (Clayton et al., 2023; Demaria et al., 2014, 2017). Thus, to selectively clear senescent cells, we incubated carotid arteries isolated from old vehicle‐ and fisetin‐supplemented p16‐3MR mice with 5 μM GCV and assessed EDD prior to and after the incubation period. Prior to GCV, we observed that vehicle‐treated mice had lower initial EDD, and senescent cell clearance with GCV incubation increased EDD, indicating that an accumulation of cellular senescence impaired endothelial function in these arteries (peak EDD: ACh alone, 76 ± 4% vs. with GCV, 90 ± 3%, *p* = 0.005). By contrast, mice that received fisetin supplementation had high initial EDD and incubation with GCV did not further improve EDD in these arteries (peak EDD: ACh alone, 93 ± 1% vs. with GCV, 92 ± 3%, *p* = 0.71; Figure 3c and Figure S3a). These findings suggest reductions in cellular senescence in arteries underlie improvements in EDD with intermittent fisetin supplementation.

#### 3.4.3 NO bioavailability

The production and subsequent bioavailability of the potent vasodilatory molecule NO is diminished in senescent endothelial cells and with aging (Hayashi et al., 2008). Thus, we next assessed the effects of fisetin on NO‐mediated endothelial function. To accomplish this, we assessed EDD prior to and following inhibition of NO production with the NO‐synthase inhibitor L‐NAME. Incubation with L‐NAME abolished group differences in EDD (peak EDD: vehicle, 37 ± 5% vs. fisetin, 44 ± 5%, *p* = 0.350; Figure 3d and Figure S3b), suggesting that improvements in endothelial function with fisetin were due to greater NO bioavailability. Consistent with this idea, peak NO‐mediated dilation (ACh alone [−] ACh with L‐NAME) was 40% greater in fisetin‐ versus vehicle‐supplemented mice (vehicle, 43 ± 4% vs. fisetin, 60 ± 5%, *p* < 0.001; Figure 3e). Next, to determine if improvements in vasodilation were a result of enhanced smooth muscle sensitivity to NO, we measured endothelium‐independent dilation as the vasodilatory response to sodium nitroprusside (SNP). We found no differences among groups (peak response to SNP, vehicle, 98 ± 1% vs. fisetin, 97 ± 1%, *p* = 0.856) indicating that fisetin improves vasodilation in aged mice in an endothelium‐specific manner (Figure 3f). Collectively, these data indicate that intermittent supplementation with fisetin enhances endothelial function in old mice as a result of greater NO bioavailability and not via increased vascular smooth muscle sensitivity to NO.

#### 3.4.4 Whole‐cell oxidative stress

**FIGURE 4:**
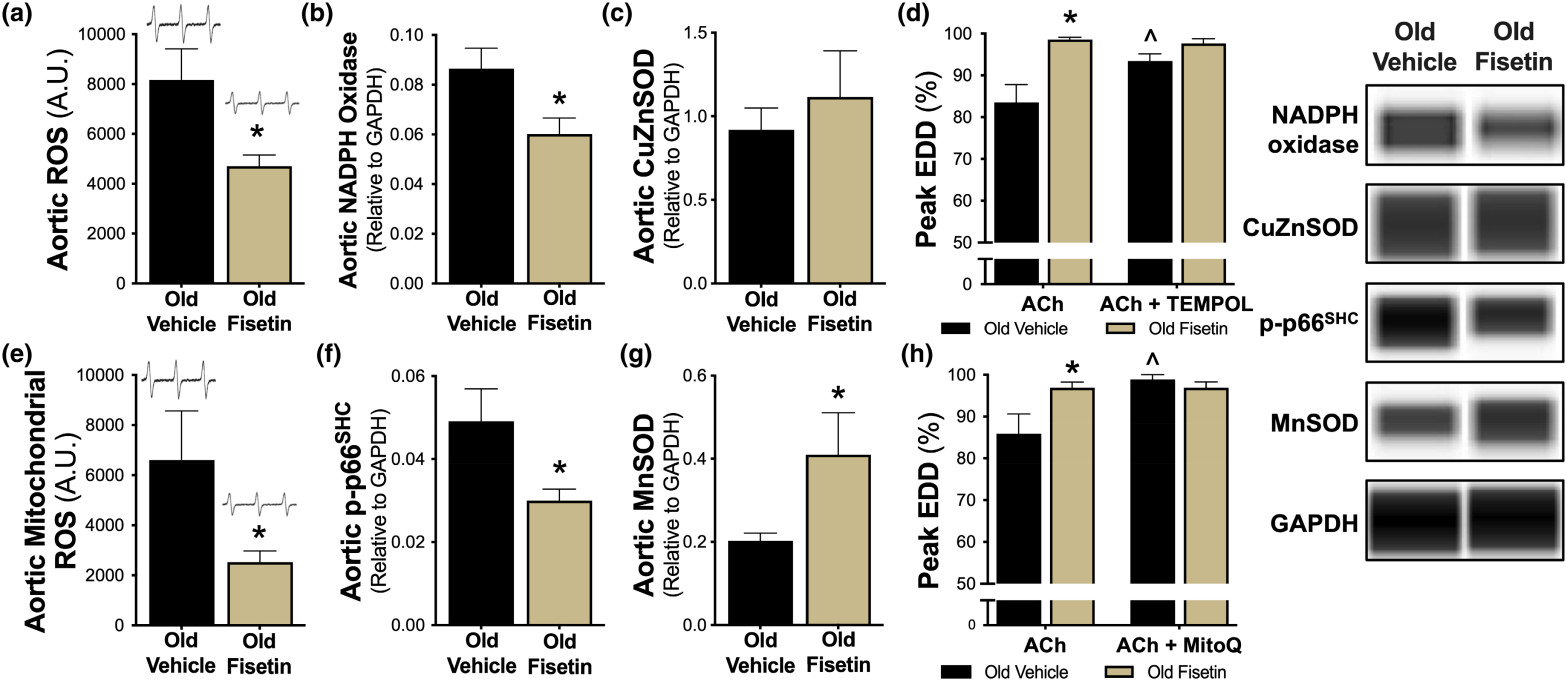
Fisetin improves endothelial function by ameliorating whole‐cell and mitochondrial oxidative stress. Whole‐cell aortic reactive oxygen species (ROS) levels (*n* = 6–8) (a). Aortic protein abundance of NADPH oxidase (*n* = 8) (b) and CuZn superoxide dismutase (SOD; *n* = 11–12) (c) with representative virtual blot bands, right. Endothelium‐dependent dilation (EDD) in isolated carotid arteries in response to acetylcholine (ACh) in the presence or absence of the SOD mimetic, TEMPOL (1 mM, 60 min preincubation; *n* = 7–8) (d). Aortic mitochondrial ROS levels (*n* = 6–10) (e). Aortic protein abundance of phosphorylated p66^SHC^ (p‐p66^SHC^; *n* = 11–12) (f) and MnSOD (*n* = 11–12) (g) with representative virtual blot bands, right. EDD in carotid arteries in response to ACh in the presence or absence of the mitochondrial‐specific antioxidant, MitoQ (1 μM; 60 min preincubation; *n* = 5–7) (h). Values represent mean ± SEM. *p < 0.05 old vehicle versus old fisetin; ^*p* < 0.05 versus ACh alone.

Age‐related reductions in NO are meditated in part by an excessive production of ROS, which react with NO reducing its bioavailability (Hsieh et al., 2014). Excess cellular senescence is associated with an increased production of ROS (Pole et al., 2016). As such, we next determined if the favorable effects of fisetin on endothelial function in old mice were associated with reduced whole‐cell oxidative stress. To do so, we first assessed ROS levels in aortic ring sections. Old fisetin‐supplemented mice had 1.7‐fold lower aortic ROS levels relative to old vehicle‐supplemented mice (vehicle, 8173 ± 1243 vs. fisetin, 4703 ± 455 arbitrary units [AU], *p* = 0.028; Figure 4a). To investigate the mechanisms responsible for lower ROS levels with fisetin supplementation, we measured the protein abundance of the pro‐oxidant enzyme NADPH oxidase, a key source of endothelial cell ROS production (Chen et al., 2008), and the cytosolic antioxidant CuZn superoxide dismutase (SOD), an important ROS scavenger (Fukai & Ushio‐Fukai, 2011), in aortic lysates. Fisetin‐supplemented mice had 30% lower NADPH oxidase abundance (vehicle, 0.086 ± 0.008 vs. fisetin, 0.060 ± 0.006 chemiluminescence units [CU], *p* = 0.027; Figure 4b and Figure S2b) but no differences in CuZnSOD abundance were observed between groups (vehicle, 0.919 ± 0.131 vs. fisetin, 1.116 ± 0.276 CU, *p* = 0.525; Figure 4c and Figure S2c). This suggests that fisetin may facilitate the reduction in tonic oxidative stress in old mice by reducing key sources of ROS which, once lowered, were in balance with cellular antioxidant status. To determine if this reduction in whole‐cell ROS levels played a direct role in the enhanced endothelial function of fisetin‐supplemented mice, we assessed EDD in isolated carotid arteries with and without the presence of the ROS scavenger TEMPOL. We found that preincubation with TEMPOL increased EDD in old vehicle‐treated mice (peak EDD: ACh alone, 84 ± 4% vs. with TEMPOL, 93 ± 2%, *p* = 0.011) toward levels observed in old fisetin‐supplemented mice with ACh alone. By contrast, the addition of TEMPOL had no effect on EDD in old fisetin‐supplemented mice (peak EDD: ACh alone, 99 ± 1% vs. with TEMPOL, 98 ± 1%, *p* = 0.487; Figure 4d and Figure S3c). Taken together, these observations indicate that fisetin improved EDD in old mice by ameliorating tonic ROS‐related suppression of EDD.

#### 3.4.5 Mitochondrial oxidative stress

Mitochondria are a primary source of excess ROS with aging and mitochondrial‐derived ROS levels are higher in senescent cells (Passos et al., 2007). Thus, we aimed to determine if fisetin supplementation reduced mitochondrial‐derived ROS in the vasculature of old mice. Fisetin‐supplemented old mice had 2.6‐fold lower aortic mitochondrial ROS levels relative to old vehicle‐supplemented mice (vehicle, 6603 ± 1956 vs. fisetin, 2527 ± 440 AU, *p* = 0.011; Figure 4e). To determine the mechanisms contributing to lower mitochondrial ROS levels with fisetin supplementation, we measured protein abundance of the activated form of the mitochondrial pro‐oxidant marker and master regulator of mitochondrial ROS production, phosphorylated p66^SHC^ (p‐p66^SHC^) (Pole et al., 2016), and the major mitochondrial antioxidant enzyme, MnSOD (Fukai & Ushio‐Fukai, 2011), in aortic lysates. Old fisetin‐supplemented mice had ~40% lower arterial abundance of p‐p66^SHC^ (vehicle, 0.049 ± 0.008 vs. fisetin, 0.030 ± 0.003 CU, *p* = 0.034; Figure 4f and Figure S2d) whereas no difference was observed in total p66^SHC^ abundance (vehicle, 0.050 ± 0.001 vs. fisetin, 0.050 ± 0.001 CU, *p* = 0.961; Figure S2e and Figure S4). MnSOD abundance was ~100% higher in old fisetin‐supplemented mice relative to old mice supplemented with the vehicle (vehicle, 0.202 ± 0.018 vs. fisetin, 0.410 ± 0.101 CU, *p* = 0.046; Figure 4g and Figure S2f). These data suggest that fisetin may reduce mitochondrial‐mediated oxidative stress by suppressing a mitochondrial pro‐oxidant enzyme and upregulating mitochondrial antioxidant defenses. To determine if lower mitochondrial ROS had a functional role in the enhanced EDD observed in old fisetin‐supplemented mice, we assessed EDD in isolated carotid arteries with and without prior incubation with the mitochondrial ROS scavenger MitoQ. We found that preincubation with MitoQ increased EDD in the old vehicle‐treated mice (peak EDD: ACh alone, 86 ± 5% vs. with MitoQ, 99 ± 1%, *p* = 0.025) to levels observed in the fisetin‐supplemented mice with ACh alone, indicating that old vehicle‐treated mice experienced tonic inhibition of EDD by excess arterial mitochondrial ROS. Importantly, we did not observe an additional increase in EDD with MitoQ in the fisetin‐supplemented group, indicating that fisetin enhanced endothelial function in old mice by ameliorating mitochondrial ROS‐related suppression of EDD (peak EDD: ACh alone, 97 ± 1% vs. with MitoQ, 97 ± 1%, *p* = 0.487; Figure 4h and Figure S3d). Collectively, these data suggests that fisetin improved EDD in old mice by ameliorating mitochondrial ROS‐related suppression of EDD.

### 3.5 Fisetin reduces aortic stiffness in old mice: Effects on intrinsic mechanical wall stiffness and components of the arterial wall

#### 3.5.1 Aortic PWV

**FIGURE 5:**
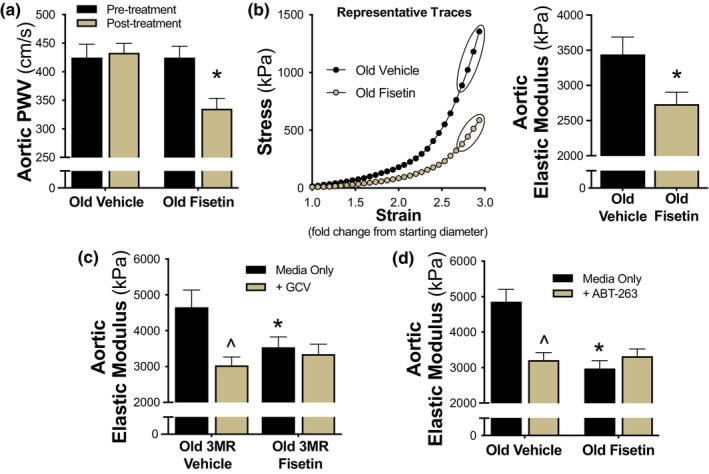
Fisetin reduces arterial stiffness in old mice by improving aortic intrinsic mechanical wall stiffness. Aortic pulse wave velocity (PWV) was measured pre‐ and post‐intervention (*n* = 9–10) (a). Representative stress–strain curve for determination of ex vivo intrinsic mechanical wall stiffness which was assessed by elastic modulus in aortic rings (calculated as the slope of the final four points in the stress–strain curve) (*n* = 7) (b). Elastic modulus was assessed after ex vivo genetic (GCV) (*n* = 8–10) (c) and pharmacological (ABT‐263) (*n* = 9–10) (d) suppression of cellular senescence in aortas obtained from old p16‐3MR (3MR) and wild‐type mice, respectively. Values represent mean ± SEM. *p < 0.05 old vehicle versus old fisetin; ^*p* < 0.05 versus media only.

To determine whether intermittent supplementation with fisetin lowers aortic stiffness in old mice, we serially assessed aortic PWV, an established analogous measure to the reference standard carotid‐femoral PWV in humans, at baseline and after treatment in both the vehicle‐ and fisetin‐supplemented mice. Aortic PWV was matched at baseline in the two groups (*p* = 0.586). Fisetin supplementation lowered aortic PWV by ~20% (pre‐, 425 ± 7 cm/s vs. post‐, 335 ± 6 cm/s, *p* < 0.001), whereas no significant change was observed over time in the vehicle‐supplemented group (pre‐, 425 ± 7 cm/s vs. post‐, 433 ± 5 cm/s, *p =* 0.403; Figure 5a). These effects occurred without obvious changes in systolic or diastolic (tail cuff) blood pressures (Table 1 and Table S1), which is in line with previous studies suggesting that age‐related aortic stiffness can be reduced independent of changes in blood pressure (Clayton, Brunt, et al., 2021; Gioscia‐Ryan et al., 2018).

#### 3.5.2 Intrinsic mechanical wall stiffness (elastic modulus)

After establishing reductions in aortic PWV with intermittent fisetin supplementation in vivo, we sought to determine whether structural modifications to the arterial wall may have contributed to this effect. To do so, we measured the elastic modulus of aortic rings isolated from old vehicle‐ and fisetin‐supplemented mice. Elastic modulus, defined as the association between the change in stress on the arterial wall in response to an increase in strain (stretch), is a key mechanism underlying increases in PWV with aging (Fleenor et al., 2012, 2013). Because this measure is conducted ex vivo, it cannot be influenced by humoral factors or blood pressure, allowing us to directly assess the influence of intermittent fisetin supplementation on arterial wall mechanical stiffness. Figure 5b shows a representative stress–strain curve of the aortic elastic modulus from old vehicle‐ and fisetin‐supplemented mice. Aortic elastic modulus was ~20% lower in old fisetin‐ versus vehicle‐supplemented mice (vehicle, 3342 ± 246 kPa vs. fisetin, 2736 ± 170 kPa, *p* = 0.012; Figure 5b) indicating that fisetin reduced the intrinsic mechanical wall stiffness of the aorta.

#### 3.5.3 Cellular senescence‐mediated intrinsic mechanical wall stiffness

To determine if fisetin reduced aortic wall stiffness by targeting cellular senescence, we used a “pharmacodissection” approach, as performed previously by our laboratory (Clayton, Brunt, et al., 2021; LaRocca et al., 2014). Here, elastic modulus was measured in aorta rings isolated from old vehicle‐ and fisetin‐supplemented p16‐3MR mice after a 48 h incubation in standard culture (control) media with or without 5 μM GCV. In aorta rings from old vehicle‐treated mice, GCV‐incubated aortic rings had a lower elastic modulus compared to aorta rings incubated in control media, indicating that cellular senescence burden contributes to the intrinsic wall stiffness of the aorta (media only, 4653 ± 482 kPa vs. GCV, 3036 ± 231 kPa, *p* = 0.009; Figure 5c). After incubation in control media, fisetin‐supplemented mice had lower initial elastic modulus compared to old vehicle‐treated mice (vehicle, 4653 ± 482 kPa vs. fisetin, 3537 ± 289 kPa, *p* = 0.054), and there was no further reduction following genetic senescent cell clearance with GCV, suggesting that fisetin effectively cleared excess vascular senescent cells to lower aortic stiffness (fisetin, media only, 3537 ± 289 kPa vs. GCV, 3346 ± 278 kPa, *p* = 0.640; Figure 5c). Taken together, these data suggest that cellular senescence influences arterial stiffness in old mice and that fisetin can modulate large elastic artery stiffening by reducing cellular senescence‐mediated increases in intrinsic mechanical wall stiffness.

We next used a complimentary approach to further establish senolysis as a primary mechanism of reductions in arterial stiffness with fisetin in old mice. We assessed elastic modulus in aorta rings isolated from old vehicle‐ and fisetin‐supplemented wild‐type mice after ex vivo incubation with the well‐established synthetic senolytic ABT‐263 (Cang et al., 2015; Chang et al., 2016). As with genetic senescent cell clearance, we found that clearance of senescent cells with ABT‐263 lowered the elastic modulus in aorta rings isolated from vehicle‐treated mice (vehicle, media only, 4860 ± 344 kPa vs. ABT‐263, 3210 ± 210 kPa, *p* = 0.002; Figure 5d). ABT‐263 exposure did not further reduce the elastic modulus in aorta rings obtained from fisetin‐supplemented mice (fisetin, media only, 2977 ± 216 kPa vs. ABT‐263, 3323 ± 199 kPa, *p* = 0.329; Figure 5d). Collectively, these results implicate cellular senescence as an underlying mechanism of large elastic artery stiffening in aged mice and suggest that fisetin decreases aortic intrinsic mechanical wall stiffness by reducing cellular senescence.

#### 3.5.4 Fisetin‐induced remodeling of the arterial wall

**FIGURE 6:**
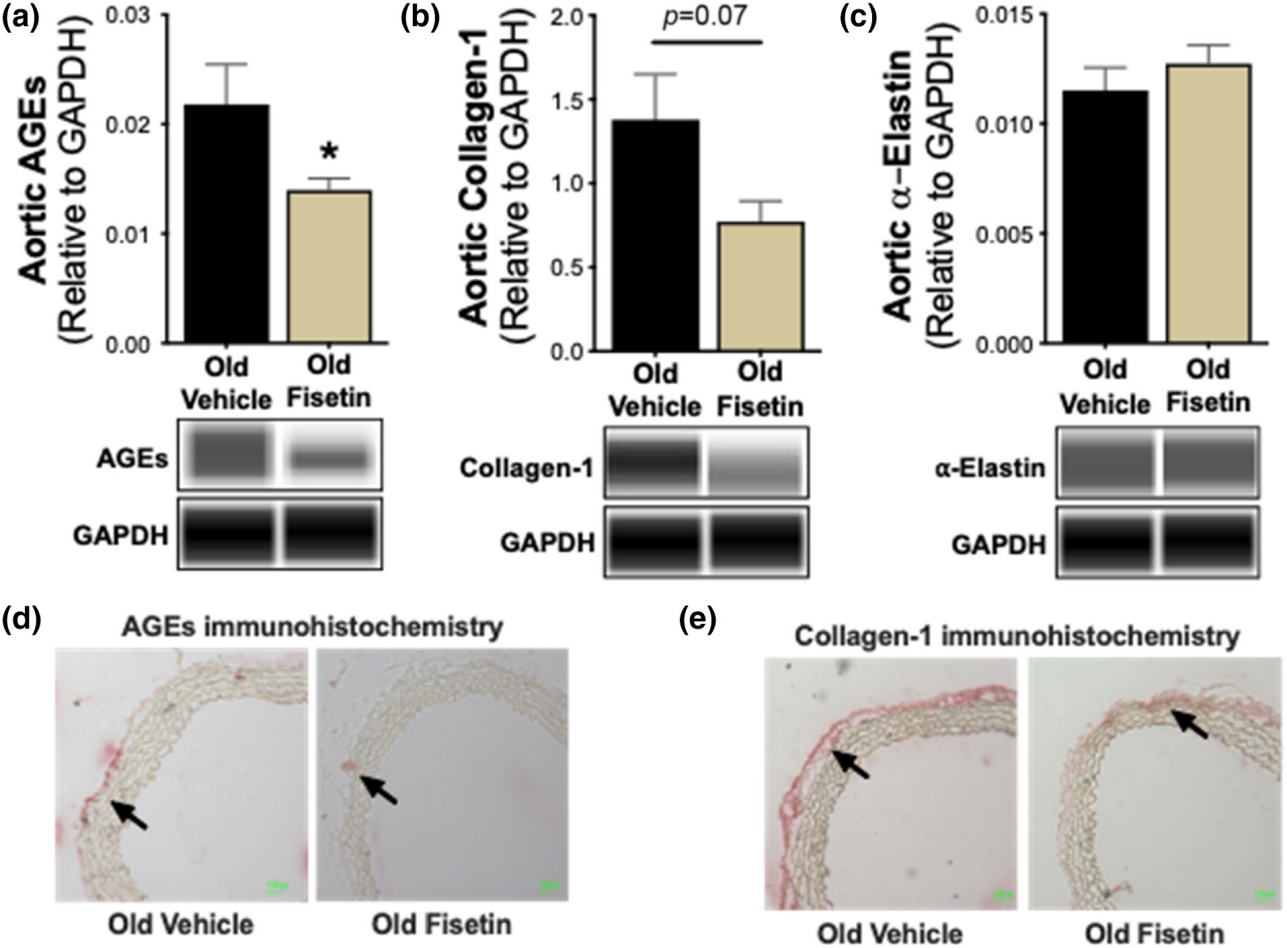
Fisetin reduces aortic intrinsic mechanical wall stiffness by favorably remodeling components of the arterial wall. Aortic protein abundance of advanced glycation end products (AGEs) (*n* = 9) (a), collagen‐1 (*n* = 9) (b), and α‐elastin (*n* = 9) (c) with representative virtual blot bands. Representative immunohistochemical staining of aortic AGEs (d) and collagen‐1 (e). Arrows denote protein accumulation in the medial‐adventitial layer. Values represent mean ± SEM. *p < 0.05 old vehicle versus old fisetin.

To gain insight into the downstream mechanisms by which fisetin reduces arterial stiffness, we measured the aortic abundance of AGEs and structural proteins using immunoblotting. The abundance of AGEs, which increases aortic stiffening by forming crosslinks in structural proteins (Zieman et al., 2005), was 36% lower in aortas from fisetin‐ versus vehicle‐supplemented mice (vehicle, 0.022 ± 0.004 vs. fisetin, 0.014 ± 0.001 CU, *p* = 0.030; Figure 6a and Figure S2g). We also observed a reduction in the major arterial isoform of collagen (collagen‐1), a protein that provides stiffness to the aortic wall (vehicle, 1.379 ± 0.271 vs. fisetin, 0.772 ± 0.123 CU, *p* = 0.070; Figure 6b and Figure S2h). By contrast, whole aorta protein abundance of α‐elastin, the primary structural protein conferring the elasticity of the arterial wall, was unaffected by fisetin treatment (vehicle, 0.012 ± 0.001 vs. fisetin, 0.013 ± 0.001 CU, *p* = 0.379; Figure 6c and Figure S2i). To gain insight into the location of these structural changes within the arterial wall, we performed immunohistochemistry on aorta sections. Reductions in the abundance of AGEs and collagen‐1 appeared to be specific to the adventitial region of the aortic wall, which is the primary load‐bearing component of the arteries under physiological conditions (Figure 6d,e). Together, these data suggest that intermittent fisetin supplementation may reduce aortic stiffness in part by favorably modulating components of the arterial wall.

## 4 DISCUSSION

Our findings demonstrate that fisetin reduces vascular cell senescence and improves arterial function in old mice. Using cell culture models, we first identified a concentration of fisetin that reduced cellular senescence in late‐passaged endothelial cells without affecting nonsenescent cells. In old mice, we next found that oral intermittent fisetin supplementation lowered markers of cellular senescence and SASP factors in the vasculature. Fisetin supplementation also improved two key manifestations of age‐related arterial dysfunction, vascular endothelial dysfunction and arterial stiffness, at least in part, by decreasing cellular senescence. Further, we found that fisetin improved vascular endothelial function by increasing NO bioavailability and reducing whole cell and mitochondrial ROS bioactivity and that reductions in arterial stiffness were accompanied with favorable remodeling of the arterial wall. These findings identify oral intermittent fisetin supplementation as a promising therapeutic strategy for clinical translation to improve arterial function with aging.

### 4.1 Fisetin and vascular cell senescence.

Cellular senescence and the SASP increase with advancing age in the vasculature (Abdellatif et al., 2023; El Assar et al., 2012; Mahoney et al., 2023; Yousefzadeh et al., 2020). Fisetin has been shown to reduce cellular senescence in fibroblasts and endothelial cells brought to senescence via ionizing radiation in culture (Zhu et al., 2017) or primary fibroblasts from progeroid mice (Yousefzadeh et al., 2018). In vivo, fisetin supplementation reduced markers of cellular senescence in various tissues in old mice (Yousefzadeh et al., 2018) and other mammalian species (Huard et al., 2023). Here, we have extended these observations and showed that fisetin at a concentration of 1 μM reduced viability of HUVECs brought to senescence via replicative exhaustion, with minimal effects in nonsenescent cells. This concentration is lower than previous concentrations shown to reduce senescent cell viability in culture (Zhu et al., 2017), an observation likely related to differences in the induction of cellular senescence. We further showed that 1 μM fisetin reduced multiple markers of cellular senescence in venous and arterial cells in vitro, establishing the senolytic effects of this concentration.

We then translated our findings to an in vivo setting using an oral dose of 100 mg/kg/day given that pharmacokinetic studies demonstrate peak plasma levels reach ~1 μM following oral ingestion of fisetin at this dose (Touil et al., 2011). We selected an established intermittent dosing paradigm for oral supplementation to remove excess senescent cells but not chronically inhibit cellular senescence activation and interfere with its principal physiological roles, and to distinguish between the senolytic effects of fisetin and any off‐target/acute effects of the compound on arterial function (Yousefzadeh et al., 2018). Consistent with our findings in endothelial cell culture, oral intermittent fisetin supplementation in vivo lowered vascular cell senescence and SASP markers in old mice.

### 4.2 Fisetin and vascular endothelial function

Vascular endothelial dysfunction is a key manifestation of age‐related arterial dysfunction and a major antecedent to overt CVDs, including atherosclerosis and occlusive stroke (Benjamin et al., 2018). In the current study, we showed that intermittent supplementation with the emerging senolytic fisetin improved endothelial function in old wild‐type mice. To demonstrate a contributing role for reductions in cellular senescence in mediating improvements in endothelial function with fisetin, we also conducted fisetin supplementation experiments in the p16‐3MR transgenic mouse model, to show that improvements in endothelial function with fisetin were mediated, in part, by a suppression of vascular cell senescence.

Age‐associated endothelial dysfunction is largely mediated by reduced NO bioavailability, which is driven mainly by excess ROS, a key source being dysregulated mitochondria (AlGhatrif et al., 2013; Ben‐Shlomo et al., 2014; Cahill & Redmond, 2016; Lakatta & Levy, 2003; Sutton‐Tyrrell et al., 2005). Vascular cell senescence is associated with diminished NO bioavailability and increased whole cell and mitochondrial ROS‐related oxidative stress (Hayashi et al., 2008). Further, mitochondrial dysfunction, including increased ROS production, is a key feature of senescent cells (Wiley et al., 2016). We showed that fisetin‐induced improvements in endothelial function were due to improvements in NO bioavailability and a reduction in whole cell‐ and mitochondrial ROS‐related suppression of endothelial function. The reductions in whole cell and mitochondrial ROS appeared to be associated with a combination of a reduction in pro‐oxidant signaling molecules (e.g., NADPH oxidase and phosphorylated‐p66^SHC^) and an increase in antioxidant enzyme defenses (e.g., MnSOD). Collectively, these findings demonstrate that oral intermittent fisetin supplementation can target excess cellular senescence to improve NO bioavailability and reduce oxidative stress to ultimately improve vascular endothelial function in old age.

### 4.3 Fisetin and large elastic artery stiffness

Increases in large elastic artery stiffness (i.e., increased aortic PWV and intrinsic mechanical wall stiffness) with aging is a major independent predictor of age‐associated clinical CVD (Vasan et al., 2022), impaired glucose tolerance (Zheng et al., 2023), kidney dysfunction (Ford et al., 2010), cognitive impairment (Thorin‐Trescases et al., 2018), and diabetic retinopathy (An et al., 2021). Fisetin supplementation reduced arterial stiffness (aortic PWV) in old wild‐type mice by reducing aortic intrinsic mechanical wall stiffness. Using the p16‐3MR mouse model, we showed that reductions in excess cell senescence with fisetin contributed to decreased aortic intrinsic mechanical wall stiffness to reduce arterial stiffness in old mice.

Pathological remodeling of the arterial wall with aging includes: (1) greater deposition of collagen‐1, the main load‐bearing protein in the aortic wall; (2) fragmentation and degradation of elastin, the primary structural protein that confers elasticity to the arteries; and (3) increased formation and expression of AGEs, which together further promoting greater arterial stiffness (Clayton, Brunt, et al., 2021; Zieman et al., 2005). Fisetin supplementation tended to reduce levels of collagen‐1 and AGEs in the adventitial regions of the arterial wall of old mice. The combination of lower collagen‐1 and AGEs levels following fisetin supplementation, despite no changes in elastin, could confer reductions in aortic stiffness. Together, these findings provide evidence that oral intermittent fisetin supplementation leads to favorable remodeling of the arterial wall, which is associated with lower aortic stiffness, and suggests that fisetin may be a viable therapeutic strategy to translate to older adults to lower arterial stiffness.

## 5 STUDY LIMITATIONS

It is possible that some of our observed effects were independent of the senolytic actions of fisetin, as fisetin modulates a variety of cell signaling pathways and has antioxidant and anti‐inflammatory properties (Farsad‐Naeimi et al., 2018; Khan et al., 2013). However, we observed reductions in multiple markers of cellular senescence and the SASP with fisetin, our measurements in mice were made 1 week after the final dose of fisetin (i.e., long after the compound is cleared from circulation (Jo et al., 2016)), and, as cellular senescence and the SASP promote arterial inflammation and oxidative stress (Clayton et al., 2020; Hayashi et al., 2008), fisetin‐induced senolysis is likely an upstream, contributing mechanism by which fisetin exerts antioxidant and anti‐inflammatory effects (Yousefzadeh et al., 2018). We also utilized genetic clearance of excess senescent cells to demonstrate that reductions in vascular cell senescence contribute to improvements in arterial function with fisetin treatment in old mice. We recognize that there are limitations to the p16‐3MR mouse model, such as elimination of nonsenescent p16^INK4A^ positive cells and limited ability to target senescent cells that do not express p16^INK4A^ (Demaria et al., 2014). Nonetheless, senescent endothelial cells highly express p16^INK4A^ and, as such, the p16‐3MR mouse model is a highly relevant model to study the role of cellular senescence in arterial aging (Clayton et al., 2023).

## 6 CONCLUSIONS

Overall, our findings provide the first evidence that oral intermittent fisetin supplementation reverses vascular endothelial dysfunction and large elastic artery stiffness through the suppression of excess cellular senescence, inflammation, and oxidative stress. Although fisetin is found in a variety of fruits and vegetables, fisetin‐rich diets are not feasible due to content variations in food. Nonetheless, fisetin is commercially available as a dietary supplement and is reported to be safe for human consumption (Bondonno et al., 2019; Farsad‐Naeimi et al., 2018; Hejazi et al., 2020; Kim & Je, 2017; Wang et al., 2019). As such, our preclinical findings provide the necessary proof‐of‐concept evidence of efficacy supporting the need for future clinical studies assessing the potential of fisetin to treat arterial dysfunction in older adults and to potentially reduce CVD risk with aging.

## AUTHOR CONTRIBUTIONS.

SAM, JC, SM, DRS, MJR, and ZSC designed the study. SAM, RV, MAD, KRL, NSV, NTG, AGL, DAH, VEB and ZSC performed the experiments. SAM, KRL, and ZSC analyzed the data. SAM, DRS, MJR, and ZSC interpreted the data and wrote the manuscript. All authors read and approved the final version of the manuscript. [Correction added on 13th January 2024, after first online publication: The author contributions have been modified in this version.]

## FUNDING INFORMATION

This work was supported by National Institute of Health Grants F31 HL165885 (to SAM), K01 DK115524 (to MJR), K99 HL159241 (to ZSC), F32 HL151022 (to ZSC), R01AG055822 (to DRS and ZSC), and R01 AG055822 (to JC, SM, and DRS) and an American Heart Association award AHA 23CDA1056582 (to MJR).

## CONFLICT OF INTEREST STATEMENT

No conflicts of interest, financial, or otherwise, are declared by the authors.

## Supporting information

Appendix S1

## Data Availability

The data that support the findings of this study are available from the corresponding authors upon reasonable request.

## References

[acel14060-bib-0001] Abdellatif, M. , Rainer, P. P. , Sedej, S. , & Kroemer, G. (2023). Hallmarks of cardiovascular ageing. Nature Reviews. Cardiology, 20, 754–777. 10.1038/s41569-023-00881-3 37193857

[acel14060-bib-0002] AlGhatrif, M. , Strait, J. B. , Morrell, C. H. , Canepa, M. , Wright, J. , Elango, P. , Scuteri, A. , Najjar, S. S. , Ferrucci, L. , & Lakatta, E. G. (2013). Longitudinal trajectories of arterial stiffness and the role of blood pressure: The Baltimore longitudinal study of aging. Hypertension, 62(5), 934–941. 10.1161/HYPERTENSIONAHA.113.01445 24001897 PMC3880832

[acel14060-bib-0003] An, Y. , Yang, Y. , Cao, B. , Dong, H. , Li, A. , Zhao, W. , Ke, J. , & Zhao, D. (2021). Increased arterial stiffness as a predictor for onset and progression of diabetic retinopathy in type 2 diabetes mellitus. Journal Diabetes Research, 2021, 9124656. 10.1155/2021/9124656 PMC8486550 34604390

[acel14060-bib-0004] Benjamin, E. J. , Virani, S. S. , Callaway, C. W. , Chamberlain, A. M. , Chang, A. R. , Cheng, S. , Chiuve, S. E. , Cushman, M. , Delling, F. N. , Deo, R. , de Ferranti, S. D. , Ferguson, J. F. , Fornage, M. , Gillespie, C. , Isasi, C. R. , Jiménez, M. C. , Jordan, L. C. , Judd, S. E. , Lackland, D. , … American Heart Association Council on Epidemiology and Prevention Statistics Committee and Stroke Statistics Subcommittee . (2018). Heart disease and stroke Statistics‐2018 update: A report from the American Heart Association. Circulation, 137(12), e67–e492. 10.1161/CIR.0000000000000558 29386200

[acel14060-bib-0005] Ben‐Shlomo, Y. , Spears, M. , Boustred, C. , May, M. , Anderson, S. G. , Benjamin, E. J. , Boutouyrie, P. , Cameron, J. , Chen, C. H. , Cruickshank, J. K. , Hwang, S. J. , Lakatta, E. G. , Laurent, S. , Maldonado, J. , Mitchell, G. F. , Najjar, S. S. , Newman, A. B. , Ohishi, M. , Pannier, B. , … Wilkinson, I. B. (2014). Aortic pulse wave velocity improves cardiovascular event prediction: An individual participant meta‐analysis of prospective observational data from 17,635 subjects. Journal of the American College of Cardiology, 63(7), 636–646. 10.1016/j.jacc.2013.09.063 24239664 PMC4401072

[acel14060-bib-0006] Bondonno, N. P. , Dalgaard, F. , Kyrø, C. , Murray, K. , Bondonno, C. P. , Lewis, J. R. , Croft, K. D. , Gislason, G. , Scalbert, A. , Cassidy, A. , Tjønneland, A. , Overvad, K. , & Hodgson, J. M. (2019). Flavonoid intake is associated with lower mortality in the Danish diet cancer and health cohort. Nature Communications, 10(1), 3651. 10.1038/s41467-019-11622-x PMC6692395 31409784

[acel14060-bib-0007] Brunt, V. E. , Gioscia‐Ryan, R. A. , Richey, J. J. , Zigler, M. C. , Cuevas, L. M. , Gonzalez, A. , Vázquez‐Baeza, Y. , Battson, M. L. , Smithson, A. T. , Gilley, A. D. , Ackermann, G. , Neilson, A. P. , Weir, T. , Davy, K. P. , Knight, R. , & Seals, D. R. (2019). Suppression of the gut microbiome ameliorates age‐related arterial dysfunction and oxidative stress in mice. The Journal of Physiology, 597(9), 2361–2378. 10.1113/JP277336 30714619 PMC6487935

[acel14060-bib-0008] Cahill, P. A. , & Redmond, E. M. (2016). Vascular endothelium—gatekeeper of vessel health. Atherosclerosis, 248, 97–109. 10.1016/j.atherosclerosis.2016.03.007 26994427 PMC6478391

[acel14060-bib-0009] Campisi, J. (2013). Aging, cellular senescence, and cancer. Annual Review of Physiology, 75, 685–705. 10.1146/annurev-physiol-030212-183653 PMC4166529 23140366

[acel14060-bib-0010] Campisi, J. , & d'Adda di Fagagna, F. (2007). Cellular senescence: When bad things happen to good cells. Nature Reviews. Molecular Cell Biology, 8(9), 729–740. 10.1038/nrm2233 17667954

[acel14060-bib-0011] Cang, S. , Iragavarapu, C. , Savooji, J. , Song, Y. , & Liu, D. (2015). ABT‐199 (venetoclax) and BCL‐2 inhibitors in clinical development. Journal of Hematology & Oncology, 8, 129. 10.1186/s13045-015-0224-3 26589495 PMC4654800

[acel14060-bib-0012] Casso, A. G. , VanDongen, N. S. , Gioscia‐Ryan, R. A. , Clayton, Z. S. , Greenberg, N. T. , Ziemba, B. P. , Hutton, D. A. , Neilson, A. P. , Davy, K. P. , Seals, D. R. , & Brunt, V. E. (2022). Initiation of 3,3‐dimethyl‐1‐butanol at midlife prevents endothelial dysfunction and attenuates in vivo aortic stiffening with ageing in mice. The Journal of Physiology, 600(21), 4633–4651. 10.1113/JP283581 36111692 PMC10069444

[acel14060-bib-0013] Chang, J. , Wang, Y. , Shao, L. , Laberge, R. M. , Demaria, M. , Campisi, J. , Janakiraman, K. , Sharpless, N. E. , Ding, S. , Feng, W. , Luo, Y. , Wang, X. , Aykin‐Burns, N. , Krager, K. , Ponnappan, U. , Hauer‐Jensen, M. , Meng, A. , & Zhou, D. (2016). Clearance of senescent cells by ABT263 rejuvenates aged hematopoietic stem cells in mice. Nature Medicine, 22(1), 78–83. 10.1038/nm.4010 PMC4762215 26657143

[acel14060-bib-0014] Chen, K. , Kirber, M. T. , Xiao, H. , Yang, Y. , & Keaney, J. F. (2008). Regulation of ROS signal transduction by NADPH oxidase 4 localization. The Journal of Cell Biology, 181(7), 1129–1139. 10.1083/jcb.200709049 18573911 PMC2442210

[acel14060-bib-0015] Clayton, Z. S. , Brunt, V. E. , Hutton, D. A. , Casso, A. G. , Ziemba, B. P. , Melov, S. , Campisi, J. , & Seals, D. R. (2021). Tumor necrosis factor alpha‐mediated inflammation and remodeling of the extracellular matrix underlies aortic stiffening induced by the common chemotherapeutic agent doxorubicin. Hypertension, 77(5), 1581–1590. 10.1161/HYPERTENSIONAHA.120.16759 33719511 PMC8035245

[acel14060-bib-0016] Clayton, Z. S. , Brunt, V. E. , Hutton, D. A. , VanDongen, N. S. , D'Alessandro, A. , Reisz, J. A. , Ziemba, B. P. , & Seals, D. R. (2020). Doxorubicin‐induced oxidative stress and endothelial dysfunction in conduit arteries is prevented by mitochondrial‐specific antioxidant treatment. JACC CardioOncol, 2(3), 475–488. 10.1016/j.jaccao.2020.06.010 33073250 PMC7561020

[acel14060-bib-0017] Clayton, Z. S. , Hutton, D. A. , Brunt, V. E. , VanDongen, N. S. , Ziemba, B. P. , Casso, A. G. , Greenberg, N. T. , Mercer, A. N. , Rossman, M. J. , Campisi, J. , Melov, S. , & Seals, D. R. (2021). Apigenin restores endothelial function by ameliorating oxidative stress, reverses aortic stiffening, and mitigates vascular inflammation with aging. American Journal of Physiology. Heart and Circulatory Physiology, 321(1), H185–H196. 10.1152/ajpheart.00118.2021 34114892 PMC8321807

[acel14060-bib-0018] Clayton, Z. S. , Rossman, M. J. , Mahoney, S. A. , Venkatasubramanian, R. , Maurer, G. S. , Hutton, D. A. , VanDongen, N. S. , Greenberg, N. T. , Longtine, A. G. , Ludwig, K. R. , Brunt, V. E. , LaRocca, T. J. , Campisi, J. , Melov, S. , & Seals, D. R. (2023). Cellular senescence contributes to large elastic artery stiffening and endothelial dysfunction with aging: Amelioration with Senolytic treatment. Hypertension, 80, 2072–2087. 10.1161/HYPERTENSIONAHA.123.21392 37593877 PMC10530538

[acel14060-bib-0019] Cristofalo, V. J. , Lorenzini, A. , Allen, R. G. , Torres, C. , & Tresini, M. (2004). Replicative senescence: a critical review. Mechanisms of Ageing and Development, 125(10–11), 827–848. 10.1016/j.mad.2004.07.010 15541776

[acel14060-bib-0020] Demaria, M. , Ohtani, N. , Youssef, S. A. , Rodier, F. , Toussaint, W. , Mitchell, J. R. , Laberge, R. M. , Vijg, J. , Van Steeg, H. , Dollé, M. E. , Hoeijmakers, J. H. , de Bruin, A. , Hara, E. , & Campisi, J. (2014). An essential role for senescent cells in optimal wound healing through secretion of PDGF‐AA. Developmental Cell, 31(6), 722–733. 10.1016/j.devcel.2014.11.012 25499914 PMC4349629

[acel14060-bib-0021] Demaria, M. , O'Leary, M. N. , Chang, J. , Shao, L. , Liu, S. , Alimirah, F. , Koenig, K. , Le, C. , Mitin, N. , Deal, A. M. , Alston, S. , Academia, E. C. , Kilmarx, S. , Valdovinos, A. , Wang, B. , de Bruin, A. , Kennedy, B. K. , Melov, S. , Zhou, D. , … Campisi, J. (2017). Cellular senescence promotes adverse effects of chemotherapy and cancer relapse. Cancer Discovery, 7(2), 165–176. 10.1158/2159-8290.CD-16-0241 27979832 PMC5296251

[acel14060-bib-0022] El Assar, M. , Angulo, J. , Vallejo, S. , Peiró, C. , Sánchez‐Ferrer, C. F. , & Rodríguez‐Mañas, L. (2012). Mechanisms involved in the aging‐induced vascular dysfunction. Frontiers in Physiology, 3, 132. 10.3389/fphys.2012.00132 22783194 PMC3361078

[acel14060-bib-0023] Farsad‐Naeimi, A. , Alizadeh, M. , Esfahani, A. , & Darvish Aminabad, E. (2018). Effect of fisetin supplementation on inflammatory factors and matrix metalloproteinase enzymes in colorectal cancer patients. Food & Function, 9(4), 2025–2031. 10.1039/c7fo01898c 29541713

[acel14060-bib-0024] Fleenor, B. S. , Seals, D. R. , Zigler, M. L. , & Sindler, A. L. (2012). Superoxide‐lowering therapy with TEMPOL reverses arterial dysfunction with aging in mice. Aging Cell, 11(2), 269–276. 10.1111/j.1474-9726.2011.00783.x 22168264 PMC3409251

[acel14060-bib-0025] Fleenor, B. S. , Sindler, A. L. , Marvi, N. K. , Howell, K. L. , Zigler, M. L. , Yoshizawa, M. , & Seals, D. R. (2013). Curcumin ameliorates arterial dysfunction and oxidative stress with aging. Experimental Gerontology, 48(2), 269–276. 10.1016/j.exger.2012.10.008 23142245 PMC3557759

[acel14060-bib-0026] Ford, M. L. , Tomlinson, L. A. , Chapman, T. P. , Rajkumar, C. , & Holt, S. G. (2010). Aortic stiffness is independently associated with rate of renal function decline in chronic kidney disease stages 3 and 4. Hypertension, 55(5), 1110–1115. 10.1161/HYPERTENSIONAHA.109.143024 20212269

[acel14060-bib-0027] Fukai, T. , & Ushio‐Fukai, M. (2011). Superoxide dismutases: Role in redox signaling, vascular function, and diseases. Antioxidants & Redox Signaling, 15(6), 1583–1606. 10.1089/ars.2011.3999 21473702 PMC3151424

[acel14060-bib-0028] Gioscia‐Ryan, R. A. , Battson, M. L. , Cuevas, L. M. , Eng, J. S. , Murphy, M. P. , & Seals, D. R. (2018). Mitochondria‐targeted antioxidant therapy with MitoQ ameliorates aortic stiffening in old mice. Journal of Applied Physiology (Bethesda, MD: 1985), 124(5), 1194–1202. 10.1152/japplphysiol.00670.2017 29074712 PMC6008077

[acel14060-bib-0029] González‐Gualda, E. , Baker, A. G. , Fruk, L. , & Muñoz‐Espín, D. (2021). A guide to assessing cellular senescence in vitro and in vivo. The FEBS Journal, 288(1), 56–80. 10.1111/febs.15570 32961620

[acel14060-bib-0030] Hayashi, T. , Yano, K. , Matsui‐Hirai, H. , Yokoo, H. , Hattori, Y. , & Iguchi, A. (2008). Nitric oxide and endothelial cellular senescence. Pharmacology & Therapeutics, 120(3), 333–339. 10.1016/j.pharmthera.2008.09.002 18930078

[acel14060-bib-0031] Hayflick, L. , & Moorhead, P. S. (1961). The serial cultivation of human diploid cell strains. Experimental Cell Research, 25, 585–621. 10.1016/0014-4827(61)90192-6 13905658

[acel14060-bib-0032] Hejazi, J. , Ghanavati, M. , Hejazi, E. , Poustchi, H. , Sepanlou, S. G. , Khoshnia, M. , Gharavi, A. , Sohrabpour, A. A. , Sotoudeh, M. , Dawsey, S. M. , Boffetta, P. , Abnet, C. C. , Kamangar, F. , Etemadi, A. , Pourshams, A. , FazeltabarMalekshah, A. , Brennan, P. , Malekzadeh, R. , & Hekmatdoost, A. (2020). Habitual dietary intake of flavonoids and all‐cause and cause‐specific mortality: Golestan cohort study. Nutrition Journal, 19(1), 108. 10.1186/s12937-020-00627-8 32988395 PMC7523365

[acel14060-bib-0033] Hickson, L. J. , Langhi Prata, L. G. P. , Bobart, S. A. , Evans, T. K. , Giorgadze, N. , Hashmi, S. K. , Herrmann, S. M. , Jensen, M. D. , Jia, Q. , Jordan, K. L. , Kellogg, T. A. , Khosla, S. , Koerber, D. M. , Lagnado, A. B. , Lawson, D. K. , LeBrasseur, N. K. , Lerman, L. O. , McDonald, K. M. , McKenzie, T. J. , … Kirkland, J. L. (2019). Senolytics decrease senescent cells in humans: Preliminary report from a clinical trial of Dasatinib plus quercetin in individuals with diabetic kidney disease. eBioMedicine, 47, 446–456. 10.1016/j.ebiom.2019.08.069 31542391 PMC6796530

[acel14060-bib-0034] Hsieh, H. J. , Liu, C. A. , Huang, B. , Tseng, A. H. , & Wang, D. L. (2014). Shear‐induced endothelial mechanotransduction: The interplay between reactive oxygen species (ROS) and nitric oxide (NO) and the pathophysiological implications. Journal of Biomedical Science, 21(1), 3. 10.1186/1423-0127-21-3 24410814 PMC3898375

[acel14060-bib-0035] Huard, C. A. , Gao, X. , Dey Hazra, M. E. , Dey Hazra, R. O. , Lebsock, K. , Easley, J. T. , Millett, P. J. , & Huard, J. (2023). Effects of Fisetin treatment on cellular senescence of various tissues and organs of old sheep. Antioxidants (Basel), 12(8), 1646. 10.3390/antiox12081646 37627641 PMC10451965

[acel14060-bib-0036] Jia, G. , Aroor, A. R. , Jia, C. , & Sowers, J. R. (2019). Endothelial cell senescence in aging‐related vascular dysfunction. Biochimica et Biophysica Acta ‐ Molecular Basis of Disease, 1865(7), 1802–1809. 10.1016/j.bbadis.2018.08.008 31109450

[acel14060-bib-0037] Jo, J. H. , Jo, J. J. , Lee, J. M. , & Lee, S. (2016). Identification of absolute conversion to geraldol from fisetin and pharmacokinetics in mouse. Journal of Chromatography. B, Analytical Technologies in the Biomedical and Life Sciences, 1038, 95–100. 10.1016/j.jchromb.2016.10.034 27810278

[acel14060-bib-0038] Justice, J. N. , Nambiar, A. M. , Tchkonia, T. , LeBrasseur, N. K. , Pascual, R. , Hashmi, S. K. , Prata, L. , Masternak, M. M. , Kritchevsky, S. B. , Musi, N. , & Kirkland, J. L. (2019). Senolytics in idiopathic pulmonary fibrosis: Results from a first‐in‐human, open‐label, pilot study. eBioMedicine, 40, 554–563. 10.1016/j.ebiom.2018.12.052 30616998 PMC6412088

[acel14060-bib-0039] Khan, N. , Syed, D. N. , Ahmad, N. , & Mukhtar, H. (2013). Fisetin: a dietary antioxidant for health promotion. Antioxidants & Redox Signaling, 19(2), 151–162. 10.1089/ars.2012.4901 23121441 PMC3689181

[acel14060-bib-0040] Kim, Y. , & Je, Y. (2017). Flavonoid intake and mortality from cardiovascular disease and all causes: A meta‐analysis of prospective cohort studies. Clinical Nutrition ESPEN, 20, 68–77. 10.1016/j.clnesp.2017.03.004 29072172

[acel14060-bib-0041] Kuehnemann, C. , Hughes, J. B. , Desprez, P. Y. , Melov, S. , Wiley, C. D. , & Campisi, J. (2023). Antiretroviral protease inhibitors induce features of cellular senescence that are reversible upon drug removal. Aging Cell, 22(1), e13750. 10.1111/acel.13750 36539941 PMC9835573

[acel14060-bib-0042] Kurz, D. J. , Decary, S. , Hong, Y. , & Erusalimsky, J. D. (2000). Senescence‐associated (beta)‐galactosidase reflects an increase in lysosomal mass during replicative ageing of human endothelial cells. Journal of Cell Science, 113(Pt 20), 3613–3622. 10.1242/jcs.113.20.3613 11017877

[acel14060-bib-0043] Lakatta, E. G. , & Levy, D. (2003). Arterial and cardiac aging: Major shareholders in cardiovascular disease enterprises: Part I: Aging arteries: a "set up" for vascular disease. Circulation, 107(1), 139–146. 10.1161/01.cir.0000048892.83521.58 12515756

[acel14060-bib-0044] LaRocca, T. J. , Hearon, C. M. , Henson, G. D. , & Seals, D. R. (2014). Mitochondrial quality control and age‐associated arterial stiffening. Experimental Gerontology, 58, 78–82. 10.1016/j.exger.2014.07.008 25034910 PMC4252265

[acel14060-bib-0045] Laurent, S. , Katsahian, S. , Fassot, C. , Tropeano, A. I. , Gautier, I. , Laloux, B. , & Boutouyrie, P. (2003). Aortic stiffness is an independent predictor of fatal stroke in essential hypertension. Stroke, 34(5), 1203–1206. 10.1161/01.STR.0000065428.03209.64 12677025

[acel14060-bib-0046] Lesniewski, L. A. , Durrant, J. R. , Connell, M. L. , Folian, B. J. , Donato, A. J. , & Seals, D. R. (2011). Salicylate treatment improves age‐associated vascular endothelial dysfunction: Potential role of nuclear factor kappaB and forkhead box O phosphorylation. The Journals of Gerontology. Series A, Biological Sciences and Medical Sciences, 66(4), 409–418. 10.1093/gerona/glq233 21303813 PMC3055281

[acel14060-bib-0047] Mahoney, S. A. , Dey, A. K. , Basisty, N. , & Herman, A. B. (2023). Identification and functional analysis of senescent cells in the cardiovascular system using omics approaches. American Journal of Physiology. Heart and Circulatory Physiology, 325(5), H1039–H1058. 10.1152/ajpheart.00352.2023 37656130 PMC10908411

[acel14060-bib-0048] National Research Council (US) Committee for the Update of the Guide for the Care and Use of Laboratory Animals . (2011). The guide for the care and use of laboratory animals (8th ed.). National Academies Press (US). 21595115

[acel14060-bib-0049] Passos, J. F. , Saretzki, G. , Ahmed, S. , Nelson, G. , Richter, T. , Peters, H. , Wappler, I. , Birket, M. J. , Harold, G. , Schaeuble, K. , Birch‐Machin, M. A. , Kirkwood, T. B. , & von Zglinicki, T. (2007). Mitochondrial dysfunction accounts for the stochastic heterogeneity in telomere‐dependent senescence. PLoS Biology, 5(5), e110. 10.1371/journal.pbio.0050110 17472436 PMC1858712

[acel14060-bib-0050] Pole, A. , Dimri, M. , & Dimri, G. P. (2016). Oxidative stress, cellular senescence and aging. AIMS Molecular Science, 3, 300–324.

[acel14060-bib-0051] Roos, C. M. , Zhang, B. , Palmer, A. K. , Ogrodnik, M. B. , Pirtskhalava, T. , Thalji, N. M. , Hagler, M. , Jurk, D. , Smith, L. A. , Casaclang‐Verzosa, G. , Zhu, Y. , Schafer, M. J. , Tchkonia, T. , Kirkland, J. L. , & Miller, J. D. (2016). Chronic senolytic treatment alleviates established vasomotor dysfunction in aged or atherosclerotic mice. Aging Cell, 15(5), 973–977. 10.1111/acel.12458 26864908 PMC5013022

[acel14060-bib-0052] Rossman, M. J. , Gioscia‐Ryan, R. A. , Clayton, Z. S. , Murphy, M. P. , & Seals, D. R. (2020). Targeting mitochondrial fitness as a strategy for healthy vascular aging. Clinical Science (London, England), 134(12), 1491–1519. 10.1042/CS20190559 32584404

[acel14060-bib-0053] Rossman, M. J. , Kaplon, R. E. , Hill, S. D. , McNamara, M. N. , Santos‐Parker, J. R. , Pierce, G. L. , Seals, D. R. , & Donato, A. J. (2017). Endothelial cell senescence with aging in healthy humans: Prevention by habitual exercise and relation to vascular endothelial function. American Journal of Physiology. Heart and Circulatory Physiology, 313(5), H890–H895. 10.1152/ajpheart.00416.2017 28971843 PMC5792201

[acel14060-bib-0054] Seals, D. R. , Jablonski, K. L. , & Donato, A. J. (2011). Aging and vascular endothelial function in humans. Clinical Science (London, England), 120(9), 357–375. 10.1042/CS20100476 PMC3482987 21244363

[acel14060-bib-0055] Steven, S. , Frenis, K. , Oelze, M. , Kalinovic, S. , Kuntic, M. , Bayo Jimenez, M. T. , Vujacic‐Mirski, K. , Helmstädter, J. , Kröller‐Schön, S. , Münzel, T. , & Daiber, A. (2019). Vascular inflammation and oxidative stress: Major triggers for cardiovascular disease. Oxidative Medicine and Cellular Longevity, 2019, 7092151. 10.1155/2019/7092151 31341533 PMC6612399

[acel14060-bib-0056] Sutton‐Tyrrell, K. , Najjar, S. S. , Boudreau, R. M. , Venkitachalam, L. , Kupelian, V. , Simonsick, E. M. , Havlik, R. , Lakatta, E. G. , Spurgeon, H. , Kritchevsky, S. , Pahor, M. , Bauer, D. , Newman, A. , & Health ABC Study . (2005). Elevated aortic pulse wave velocity, a marker of arterial stiffness, predicts cardiovascular events in well‐functioning older adults. Circulation, 111(25), 3384–3390. 10.1161/CIRCULATIONAHA.104.483628 15967850

[acel14060-bib-0057] Thorin‐Trescases, N. , de Montgolfier, O. , Pinçon, A. , Raignault, A. , Caland, L. , Labbé, P. , & Thorin, E. (2018). Impact of pulse pressure on cerebrovascular events leading to age‐related cognitive decline. American Journal of Physiology. Heart and Circulatory Physiology, 314(6), H1214–H1224. 10.1152/ajpheart.00637.2017 29451817 PMC6032083

[acel14060-bib-0058] Touil, Y. S. , Auzeil, N. , Boulinguez, F. , Saighi, H. , Regazzetti, A. , Scherman, D. , & Chabot, G. G. (2011). Fisetin disposition and metabolism in mice: Identification of geraldol as an active metabolite. Biochemical Pharmacology, 82(11), 1731–1739. 10.1016/j.bcp.2011.07.097 21840301

[acel14060-bib-0059] Udvardi, M. K. , Czechowski, T. , & Scheible, W. R. (2008). Eleven golden rules of quantitative RT‐PCR. Plant Cell, 20(7), 1736–1737. 10.1105/tpc.108.061143 18664613 PMC2518243

[acel14060-bib-0060] van Deursen, J. M. (2014). The role of senescent cells in ageing. Nature, 509(7501), 439–446. 10.1038/nature13193 24848057 PMC4214092

[acel14060-bib-0061] Vasan, R. S. , Pan, S. , Xanthakis, V. , Beiser, A. , Larson, M. G. , Seshadri, S. , & Mitchell, G. F. (2022). Arterial stiffness and long‐term risk of health outcomes: The Framingham heart study. Hypertension, 79(5), 1045–1056. 10.1161/HYPERTENSIONAHA.121.18776 35168368 PMC9009137

[acel14060-bib-0062] Vlachopoulos, C. , Aznaouridis, K. , & Stefanadis, C. (2010). Prediction of cardiovascular events and all‐cause mortality with arterial stiffness: a systematic review and meta‐analysis. Journal of the American College of Cardiology, 55(13), 1318–1327. 10.1016/j.jacc.2009.10.061 20338492

[acel14060-bib-0063] Wang, L. , Cao, D. , Wu, H. , Jia, H. , Yang, C. , & Zhang, L. (2019). Fisetin prolongs therapy window of brain ischemic stroke using tissue plasminogen activator: A double‐blind randomized placebo‐controlled clinical trial. Clinical and Applied Thrombosis/Hemostasis, 25, 1076029619871359. 10.1177/1076029619871359 31434498 PMC6829632

[acel14060-bib-0064] Wiley, C. D. , Velarde, M. C. , Lecot, P. , Liu, S. , Sarnoski, E. A. , Freund, A. , Shirakawa, K. , Lim, H. W. , Davis, S. S. , Ramanathan, A. , Gerencser, A. A. , Verdin, E. , & Campisi, J. (2016). Mitochondrial dysfunction induces senescence with a distinct secretory phenotype. Cell Metabolism, 23(2), 303–314. 10.1016/j.cmet.2015.11.011 26686024 PMC4749409

[acel14060-bib-0065] Yousefzadeh, M. J. , Zhao, J. , Bukata, C. , Wade, E. A. , McGowan, S. J. , Angelini, L. A. , Bank, M. P. , Gurkar, A. U. , McGuckian, C. A. , Calubag, M. F. , Kato, J. I. , Burd, C. E. , Robbins, P. D. , & Niedernhofer, L. J. (2020). Tissue specificity of senescent cell accumulation during physiologic and accelerated aging of mice. Aging Cell, 19(3), e13094. 10.1111/acel.13094 31981461 PMC7059165

[acel14060-bib-0066] Yousefzadeh, M. J. , Zhu, Y. , McGowan, S. J. , Angelini, L. , Fuhrmann‐Stroissnigg, H. , Xu, M. , Ling, Y. Y. , Melos, K. I. , Pirtskhalava, T. , Inman, C. L. , McGuckian, C. , Wade, E. A. , Kato, J. I. , Grassi, D. , Wentworth, M. , Burd, C. E. , Arriaga, E. A. , Ladiges, W. L. , Tchkonia, T. , … Niedernhofer, L. J. (2018). Fisetin is a senotherapeutic that extends health and lifespan. eBioMedicine, 36, 18–28. 10.1016/j.ebiom.2018.09.015 30279143 PMC6197652

[acel14060-bib-0067] Zheng, H. , Wu, S. , Liu, X. , Qiu, G. , Chen, S. , Wu, Y. , Li, J. , Yin, C. , & Zhang, Q. (2023). Association between arterial stiffness and new‐onset heart failure: The Kailuan study. Arteriosclerosis, Thrombosis, and Vascular Biology, 43(2), e104–e111. 10.1161/ATVBAHA.122.317715 36579648

[acel14060-bib-0068] Zhu, Y. , Doornebal, E. J. , Pirtskhalava, T. , Giorgadze, N. , Wentworth, M. , Fuhrmann‐Stroissnigg, H. , Niedernhofer, L. J. , Robbins, P. D. , Tchkonia, T. , & Kirkland, J. L. (2017). New agents that target senescent cells: The flavone, fisetin, and the BCL‐X. Aging (Albany NY), 9(3), 955–963. 10.18632/aging.101202 28273655 PMC5391241

[acel14060-bib-0069] Zieman, S. J. , Melenovsky, V. , & Kass, D. A. (2005). Mechanisms, pathophysiology, and therapy of arterial stiffness. Arteriosclerosis, Thrombosis, and Vascular Biology, 25(5), 932–943. 10.1161/01.ATV.0000160548.78317.29 15731494

