# Supplementary material for: Intermittent supplementation with fisetin improves arterial function in old mice by decreasing cellular senescence
Source: Aging Cell. 2023 Dec 7;23(3):e14060. doi: 10.1111/acel.14060 (PMC10928570; doi:10.1111/acel.14060)
Supplement: Supplementary file 1 — Appendix S1 [file ACEL-23-e14060-s001.docx]

**SUPPLEMENTAL METHODS**

**Cell Culture Experiments.**

***Cell Viability Assay.***

Cell viability was assayed on a BioTek Synergy Neo2 using CellTiterGlo (Promega, Madison, WI, Cat. No. G7570) according to the manufacturer’s instructions. Briefly, cells were plated in a white-bottom 384 well plate (Corning Inc., Corning, NY) and treated with the indicated concentrations of fisetin, as described (Scott et al., 2019). Viability was assessed 48hr after treatment and normalized to the DMSO control. IC_50_ values were calculated using GraphPad Prism. All values are the mean of 4-8 replicate measurements.

***Senescence-associated β-galactosidase (SA-β-gal) staining.***

SA-β-gal staining was performed using the Senescence Detection Kit (Abcam, Cambridge, UK, Cat. No. ab65351) according to the manufacturer’s instructions. Briefly, after overnight seeding, late passaged human umbilical vein endothelial cells (HUVECs) were treated with 1μM fisetin in media for 48hr. Following the incubation period, early passaged (control), late passaged (senescent), and senescent cells treated with 1μM were washed, fixed, and incubated with the X-gal solution overnight. Images were captured using bright-field microscopy at 20X magnification and quantified using ImageJ, as described (Kurz, Decary, Hong, & Erusalimsky, 2000).

***Assessment of Reactive Oxygen Species (ROS) levels.***

Oxidative stress was detected in cells using the CellROX Deep Red ROS probe (Invitrogen, Waltham, MA) according to the manufacturer’s instructions. Briefly, cells were plated in a high content image plate with 0.2mm glass bottom (Corning, Kennebunk, ME) coated with bovine gelatin substrate (Attachment Factor Solution; Cell Applications, Inc., San Diego, CA) to facilitate cell adhesion. After a 24hr incubation, attached cells were washed once with Hanks’ balanced salt solution (HBSS) and treated with 1μM fisetin for 4hr. Attached cells were then washed twice with HBSS and incubated with 9.2μM CellROX Deep Red ROS probe for 60 minutes. After dye removal and three washes with HBSS, cells were left in 100μL of HBSS for subsequent imaging. Microscopy was carried out on an EVOS FL microscope (Invitrogen, Carlsbad, CA) set at 20X magnification. Cells were selected for imaging based on characteristic endothelial cell and nuclear morphology. Cell images were analyzed with Fiji (National Institutes of Health) and image intensity was averaged over the entire cell area (Schindelin et al., 2012).

***Cell and Aortic Gene Expression.***

mRNA gene expression was measured in human aortic endothelial cells (HAECs) and segments of thoracic aorta following mechanical homogenization. RNA was extracted using the RNeasy mini kit (Qiagen, Hilden, Germany). cDNA was synthesized using the iScript cDNA synthesis kit (Bio-Rad Laboratories, Hercules, CA). Transcripts of cellular senescence and senescence-associated secretory phenotype (SASP) markers (primer sequences reported in Table S2) were analyzed using a StepOnePlus Real-Time PCR System (Applied Biosystems, Waltham, MA) in 96-well plates and the Taqman OpenArray (Applied Biosystems, Waltham, MA) was used as a master mix, as described (Lesniewski et al., 2017). SimpleSeq DNA sequencing (Quintara Biosciences, Cambridge, MA) was used to validate PCR products.

**Animal Experiments.**

***Animals and Experimental Design.***

Male C57Bl/6N (wildtype) mice were obtained from the National Institute of Aging colony (maintained by Charles River, Wilmington, MA). Wildtype mice were allowed to acclimate to our facilities for 4 weeks before beginning the study. Male and female p16-3MR mice were bred and aged in our mouse colony at the University of Colorado Boulder. These mice carry a trimodal fusion protein (3MR) under the control of the p16^INK4A^ promoter which allows for selective genetic clearance of p16^INK4A^-positive (p16^+^) senescent cells by administering the antiviral agent ganciclovir (GCV) (Demaria et al., 2014). For the duration of the study, all mice were single housed at the University of Colorado Boulder animal facility with a 12hr:12hr light-dark cycle and allowed ad libitum access to an irradiated, fixed, and open rodent chow (Inotiv/Envigo 7917, stored at room temperature).

For the intervention period, wildtype and p16-3MR mice were randomly assigned to receive vehicle (10% EtOH, 30% PEG400 and 60% Phosal 50 PG) or fisetin (50 mg/kg/day in vehicle). For wildtype mice, 18 mice received vehicle and 19 mice received fisetin, and for p16-3MR mice, 27 mice received vehicle and 35 mice received fisetin. Treatment was administered via oral gavage using an intermittent dosing paradigm – one week on; two weeks off; one week on. Mice were sacrificed one to two weeks following the final dose to rule out any acute effects of the compound as the terminal half-life of fisetin is ~3.1 hours in plasma (Jo, Jo, Lee, & Lee, 2016). Animals were administered vehicle or fisetin daily during the treatment period. Treatment groups were matched for baseline body weight and aortic PWV. Throughout this intervention period, 1 old wildtype mouse and 11 p16-3MR mice died as a result of typical age-related attrition, which resulted in a final sample size of old Vehicle, n = 17; old Fisetin, n = 19; old 3MR Vehicle, n = 22; and old 3MR Fisetin, n = 29.

***In Vivo Aortic Stiffness and Arterial Blood Pressure.***

Aortic stiffness was assessed using the reference standard non-invasive *in vivo* measure, aortic PWV, one week before (pre) and one week after (post) the intervention, as previously described (Brunt et al., 2019; Casso et al., 2022; Clayton, Brunt, et al., 2021; Clayton, Hutton, et al., 2021). Briefly, mice were placed under light isoflurane anesthesia (1.0-2.5%) and positioned supine on a warmed heat pad. Front- and hind-limb paws were then secured to corresponding ECG electrodes. Two Doppler probes were then placed on the skin at the transverse aortic arch and the abdominal aorta. Once clear R-waves were registered, three repeated 2-second ultrasound tracings were recorded and average pre-ejection time (i.e., time between the R-wave of the ECG to the foot of the Doppler signal) was determined for each location. To calculate aortic PWV, the distance between the two probes was divided by the difference between the transverse aortic arch and abdominal aorta pre-ejection times (time_abdominal_ – time_arch_) and is reported as centimeters/second (cm/s). To examine the potential contribution of changes in arterial blood pressure to any treatment-related differences in aortic PWV, systolic and diastolic blood pressures were assessed using a CODA noninvasive tail-cuff system (Kent Scientific, Torrington, CT), as we have described previously (Brunt et al., 2019; Casso et al., 2022; Clayton, Brunt, et al., 2021; Clayton, Hutton, et al., 2021). Briefly, the pressure measurements from 20 collection cycles (following 5 acclimation cycles) for 3 consecutive days were averaged per mouse at each timepoint.

***Vascular endothelial function: endothelium-dependent dilation (EDD), cellular senescence-mediated suppression of EDD, NO-mediated EDD, whole-cell and mitochondrial-specific ROS suppression of EDD, and endothelium-independent dilation (EID).***

Upon completion of *in vivo* measures, mice were euthanized via cardiac exsanguination under inhaled isoflurane anesthesia, and carotid arteries were immediately excised. Vasodilatory function was measured via *ex vivo* carotid artery EDD and EID in response to increasing doses of acetylcholine (ACh) and sodium nitroprusside (SNP), respectively, as described previously (Brunt et al., 2019; Casso et al., 2022; Clayton, Brunt, et al., 2021; Clayton, Hutton, et al., 2021). In brief, after vessels were pre-constricted with phenylephrine (PE; 2mM; Sigma-Aldrich, Cat. No. P6126), EDD was assessed by measuring increases in luminal diameter in response to increasing concentrations of ACh (1 X 10^-9^ to 1 X 10^-4^ M; Sigma-Aldrich, Cat. No. A6625) with and without *ex vivo* coadministration of GCV (5μm, 180min pre-incubation; Sigma Aldrich, St. Louis, MO, Cat. No. G2536), tonic ROS scavenger TEMPOL (1mM, 60min pre-incubation; Sigma-Aldrich, Cat. No. 2226-96-2), mitochondrial-specific ROS scavenger MitoQ (1µM, 60min pre-incubation; Antipodean Pharmaceuticals, Inc., Menlo Park, CA, USA), or the NO synthase inhibitor *N*^G^-nitro-L-arginine methylester (L-NAME, 0.1mM, 30min pre-incubation; Sigma-Aldrich, Cat. No. N5751). NO-mediated dilation was then calculated as the difference between maximum EDD to ACh alone and in the presence of ACh and L-NAME:

NO-mediated dilation (%) = Maximum dilation_Ach_ - Maximum dilation_Ach+L-NAME_

Following EDD and pharamo-dissections of EDD, EID was assessed by measuring the increase in luminal diameter in response to increasing concentrations of SNP, an exogenous NO donor (1 X 10^-10^ to 1 X 10^-4^ M; Sigma-Aldrich, Cat. No. 13755-38-9). All dose response data are presented as percent dilation relative to maximum diameter to account for differences in baseline vessel diameter.

***Aortic ROS Production.***

Whole-cell aortic ROS production was assessed using the spin probe 1-hydroxy-3-methoxycarbonyl-2,2,5,5-tetramethylpyrrolidine (CMH; Enzo Life Sciences, Farmingdale, NY, Cat. No. ALX-430-078) and mitochondrial-specific ROS production was assessed using the spin probe 1-hydroxy-4-[2-triphenylphosphonio-acetamido]-2,2,6,6-tetramethylpiperidine (mitoTEMPO-H; Enzo Life Sciences, Cat. No. ALX-430-171-M005) by electron paramagnetic resonance (EPR) spectrometry, as previously described (Brunt et al., 2019; Casso et al., 2022; Clayton, Brunt, et al., 2021; Clayton, Hutton, et al., 2021). In short, two 1-mm aortic rings were washed in warm physiological saline solution and incubated in Krebs/HEPES buffer, consisting of 99mM NaCl, 4.7mM KCl, 1.87mM CaCl_2_, 1.2mM MgSO_4_, 25mM NaHCO_3_, 1.03mM KH_2_PO_4_, 20mM Na-HEPES, 11.1mM glucose, 0.1mM diethylenetriaminepenta-acetic acid, 0.0035mM sodium diethyldithiocarbamate, and Chelex (Sigma-Aldrich, Cat. No. C7901), containing 0.5mM CMH or mitoTEMPO-H at 37°C for 60min. Samples were analyzed using aMS300 Xband EPR spectrometer (Magnettech, Berlin, Germany) with the following instrument parameters: B0-Field, 3350G; sweep, 80G; sweep time, 60s; modulation, 3000mG; MWatten, 7dB; gain, 500.

***Aortic Intrinsic Mechanical Wall Stiffness (Elastic Modulus) and Cellular Senescence-Mediated Changes in Aortic Intrinsic Mechanical Wall Stiffness.***

Aortas were promptly excised from the mice following carotid artery excision, rinsed with cold physiological saline solution, and cleared of any remnant perivascular adipose and connective tissue. To measure *ex vivo* aortic stiffness, two thoracic aorta samples (~1 mm in length) were cut and used to determine intrinsic mechanical stiffness via pin myography as we have previously described (Casso et al., 2022; Clayton, Brunt, et al., 2021; Clayton, Hutton, et al., 2021). In short, aorta samples were placed in heated (37°C) baths filled with calcium-free, phosphate-buffered saline (PBS). The samples were then mounted on two wire prongs, followed by three rounds of pre-stretching. Once pre-stretching was complete, aortic ring diameter was increased until 1mN of force was reached and incrementally increased by 5µm every 3min thereafter until failure. The force corresponding to each stretching interval was recorded and used to calculate stress and strain. A stress-strain curve was then generated using the following equations:

where *d* is diameter and *d_i_* is initial diameter.

where *L* is one-dimensional load, *H* is intima media thickness, and *D* is vessel length.

The elastic modulus of the stress-strain curve was determined as the slope of the linear regression fit to the final four points of the stress-strain curve, as previously reported by our laboratory (Brunt et al., 2019; Casso et al., 2022; Clayton, Brunt, et al., 2021; Clayton, Hutton, et al., 2021). To assess the stiffening role of cellular senescence and the beneficial effects of fisetin on intrinsic mechanical stiffness, wildtype aorta rings were pre-incubated with 2.5µM ABT-263, a synthetic senolytic, and p16-3MR aortas were pre-incubated with 5µM GCV, which are concentrations that have been shown to reduce the abundance of senescent cells *ex vivo* (Chang et al., 2016; Demaria et al., 2017)*,* for 48hr prior to the following measurements. Aortic intima media thickness and diameter were assessed as we have described previously (Brunt et al., 2019; Casso et al., 2022; Clayton, Brunt, et al., 2021; Clayton, Hutton, et al., 2021). Briefly, aortic rings (1mm) were frozen in optimal cutting temperature solution and stored at -80°C until the time of sectioning. Aortic sectioning was performed on a cryostat (7µm; Leica CM300, Leica Biosystems, Wetzlar, Germany) at -22°C and sections were visualized, and images were captured with a bright-field microscope. Aortic intima media thickness and diameter were calculated using ImageJ software.

***Immunohistochemistry in Mouse Aortas.***

The localization of collagen-1 and advanced glycation end-products (AGEs) were assessed using ~1mm sections of mouse thoracic aorta which were collected at the time of sacrifice, frozen in OCT compound, and stored at -80°C until the time of sectioning. Samples were later sectioned (7µm; Leica CM300, Leica Biosystems, Wetzlar, Germany) and plated in poly-L-lysine coated slides, fixed in 4% paraformaldehyde and stored at -80°C until further use in immunohistochemical analyses. Upon staining, the slides were rehydrated (PBS with 50mM glycine), washed with PBS, permeabilized (0.1% Triton X-100), and incubated for 20 minutes with 2.5% normal horse serum blocking buffer following 3 washes with PBS. Slides were then incubated with primary antibodies at room temperature for 1 hour, washed 3 times with PBS, incubated with horseradish peroxidase-conjugated secondary antibody for 30 minutes, treated with 1:1 3,3’-Diaminobenzidine (DAB):H_2_O_2_ for approximately 30 minutes as the samples began to darken, and then immediately washed in PBS and cured overnight with gelvatol. Collagen-1 and AGEs primary antibodies (antibody descriptions reported in Table S3 were used for aortic protein targets, as previously described (Brunt et al., 2019; Clayton, Brunt, et al., 2021). The slides were imaged using a Nikon Eclipse TS100 under identical conditional and analyzed using ImageJ.

***Aortic Protein Abundance.***

Protein abundance was measured in segments of thoracic aorta following mechanical homogenization in radioimmunoprecipitation assay lysis buffer supplemented with protease and phosphatase inhibitors (1mM sodium orthovanadate, 1X complete mini protease inhibitor cocktail tablet [Roche, Mannheim, Germany, Cat. No. 11836153001], 1 mM phenylmethylsulphonyl fluoride, 1:100 Phosphatase Inhibitor Cocktail [Sigma-Aldrich, Cat. No. P2850], 5mM sodium fluoride, and 5mM sodium pyrophosphate). Total protein content was quantified using a bicinchoninic acid assay (Thermo Fisher Scientific, Cat. No. 23225). Next, abundance of AGEs, α-elastin, collagen-1, p16^INK4A^, total and phosphorylated p66^SHC^, nicotinamide adenine dinucleotide phosphate (NAPDH) oxidase, CuZn superoxide dismutase (SOD), MnSOD, and Glyceraldehyde 3-phosphate dehydrogenase (GAPDH) (antibody descriptions reported in Table S3) were determined by loading 20ng/mL of aortic protein per capillary in a 25-lane (capillary) automated Western blot quantitative analyzer (WES, ProteinSimple, San Jose, CA), according to the manufacturer’s guidelines, as described previously (Brunt et al., 2019; Casso et al., 2022; Clayton, Brunt, et al., 2021; Clayton, Hutton, et al., 2021), following the validation of these antibodies in test aorta lysates. Secondary antibodies were provided by the manufacturer and used according to the manufacturer’s guidelines. A grayscale analysis of the band intensities was then performed to quantify protein abundance using Compass software (ProteinSimple), with target proteins expressed relative to a loading control (GAPDH). Data files may be accessed at DOI: 10.6084/m9.figshare.24061176.

***Statistical Analyses.***

Power calculations were performed using G*power 3.1 (RRID: SCR_013726) for our primary outcome variable, aortic PWV. Previously, our laboratory has obtained effect sizes of 1.35 when comparing aortic PWV between treatment groups. With this effect size, N=6 mice per condition were required to achieve 99% statistical power. Additional mice were studied in each group to ensure sufficient PWV traces were obtained and to account for age-related attrition.

Statistical analyses were conducted using GraphPad Prism version 9.4.0 (GraphPad Software, Inc., San Diego, CA, USA; RRID:SCR_002798). Data were assessed for statistical outliers (ROUT test; Q = 1%), and outliers were excluded from final analyses. All variables were assessed using unpaired t-test Statistical significance was set to α=0.05. Data are presented as mean ± SEM

**SUPPLEMENTAL TABLES**

**Table S1.** p16-3MR Animal Characteristics

| Characteristics | Vehicle | Fisetin |
| --- | --- | --- |
| *n* | 15 | 18 |
| Body mass, g | 27.9 ± 0.7 | 27.9 ± 1.0 |
| Heart mass, mg | 177 ± 6 | 186 ± 8 |
| Quadriceps mass, mg | 179 ± 9 | 175 ± 9 |
| Visceral adipose mass, mg | 266 ± 45 | 233 ± 42 |
| Liver mass, g | 1.4 ± 0.1 | 1.6 ± 0.1 |
| Spleen mass, mg | 109 ± 27 | 100 ± 18 |
| Carotid artery |  |  |
| *Resting diameter, µm* | 428 ± 11 | 427 ± 9 |
| *Maximal diameter, µm* | 496 ± 10 | 493 ± 7 |
| Aorta |  |  |
| *Diameter, µm* | 386 ± 6 | 412 ± 13 |
| *Intima media thickness, µm* | 37 ± 2 | 33 ± 1 |
| Systolic blood pressure, mmHg |  |  |
| *Pre* | 95 ± 3 | 96 ± 2 |
| *Post* | 98 ± 3 | 96 ± 2 |
| Diastolic blood pressure, mmHg |  |  |
| *Pre* | 65 ± 3 | 66 ± 2 |
| *Post* | 69 ± 3 | 67 ± 2 |

Values represent mean ± SEM.

**Table S2.** Primer sequences.

| Gene | Species | Forward primer | Reverse primer |
| --- | --- | --- | --- |
| *Cdkn2a* | Human | GAGCAGCATGGAGCCTTC | CCGTTTTCGACCCTGAGAG |
| *Cdkn1a* | Human | TCACTGTCTTGTACCCTTGTGC | TTTGCTCCTGTGCGGAAC |
| *Gapdh* | Human | ATGTTCGTCATGGGTGTGAA | GGTGCTAAGCAGTTGGTGGT |
| *Cdkn2a* | Mouse | CCCAACGCCCCGAACT | GCAGAAGAGCTGCTACGTGAA |
| *Cdkn1a* | Mouse | TTGCCAGCAGAATAAAAGGTG | TTTGCTCCTGTGCGGAAC |
| *Serpine1* | Mouse | TGGAAGGGCAACATGACCAG | TCAGGCATGCCCAACTTCTC |
| *Lmnb1* | Mouse | GAGCCCCAAGAGCATCCAAT | CTGAGAAGGCTCTGCACTGT |
| *Cxcl2* | Mouse | CCTGGTTCAGAAAATCATCCA | CTTCCGTTGAGGGACAGC |
| *Vegf* | Mouse | AAAAACGAAAGCGCAAGAAA | TTTCTCCGCTCTGAACAAGG |
| *Cxcl1* | Mouse | CTGGGATTCACCTCAAGAACATC | CAGGGTCAAGGCAAGCCTC |
| *Tnf-α* | Mouse | ATGAGAAGTTCCCAAATGGC | CTCCACTTGGTGGTTTGCTA |
| *Mmp3* | Mouse | CTCGTGGTACCCACCAAGTC | CGCCAAAAGTGCCTGTCTTT |
| *Ccl2* | Mouse | CACTCACCTGCTGCTACTCA | GCTTGGTGACAAAAACTACAGC |
| *Plat* | Mouse | AAGCATGAGGCATCGTCTCC | ATGCATCGTGGAGGTCTTGG |
| *Gapdh* | Mouse | AAGGTCATCCCAGAGCTGAA | CTGCTTCACCACCTTCTTGA |

**Table S3.** Antibodies.

| Protein | Assay | Species | Dilution factor | Company | Catalog no. |
| --- | --- | --- | --- | --- | --- |
| Advanced glycation end-products (AGEs) | IHC | Rabbit | 1:200 | Abcam | ab23722 |
| Collagen-1 | IHC | Rabbit | 1:200 | Sigma Aldrich | 234167 |
| AGEs | Wes | Rabbit | 1:250 | Abcam | ab23722 |
| α-Elastin | Wes | Rabbit | 1:20 | Invitrogen | PA572440 |
| Collagen-1 | Wes | Rabbit | 1:10 | Sigma Aldrich | 234167 |
| p16^INK4A^ | Wes | Rabbit | 1:20 | Invitrogen | PA5-20379 |
| p66^SHC^ | Wes | Rabbit | 1:10 | Sigma Aldrich | AV50490 |
| phosphorylated-p66^SHC^ | Wes | Mouse | 1:10 | Abcam | AB54518 |
| Nicotinamide adenine dinucleotide phosphate (NAPDH) oxidase | Wes | Goat | 1:200 | BD Bioscience | 610912 |
| CuZn Superoxide dismutase (SOD) | Wes | Goat | 1:500 | R&D Systems | AF3787 |
| MnSOD | Wes | Goat | 1:50 | R&D Systems | AF3419 |
| Glyceraldehyde 3-phosphate dehydrogenase (GAPDH) | Wes | Rabbit | 1:200 | Cell Signaling | 14C10 |

IHC: Immunohistochemistry; WES: Simple Western’s WES Instrument.

**SUPPLEMENTAL FIGURES**


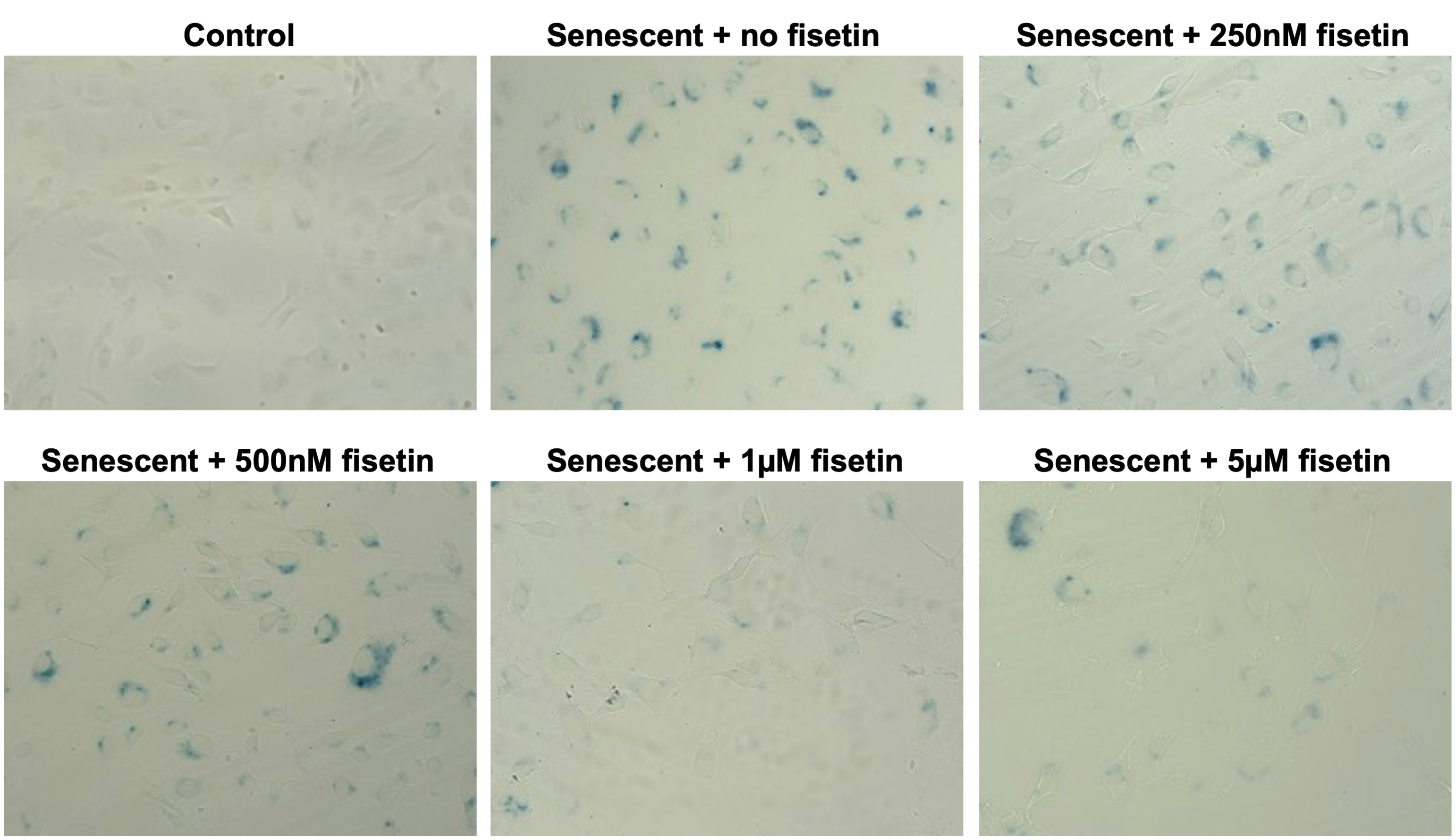


**Figure S1.** Representative images of senescence-associated beta galactosidase (SA-β-Gal) signal in non-senescent (control) and senescent human umbilical vein endothelial cells to increasing doses of fisetin.

**Figure S2.** Representative Western Blots in aorta lysates from old mice treated with vehicle (Veh) or fisetin (Fis) for the following protein targets: p16^INK4A^ (n=8) **(A)**, NADPH oxidase (n=8) **(B)**, CuZN superoxide dismutase (SOD; n=11-12) **(C)**, phosphorylated (p) p66^SHC^ (n=11-12) **(D)**, total p66^SHC^ (n=8-10) **(E)**, MnSOD (n=11=12) **(F)**, advanced glycation end-products (AGEs; n=9) **(G)**, collagen-1 (n=9) **(H)**, and elastin (n=9) **(I)**. Individual blots are separated by the protein ladder (L) and all proteins were normalized to GAPDH.
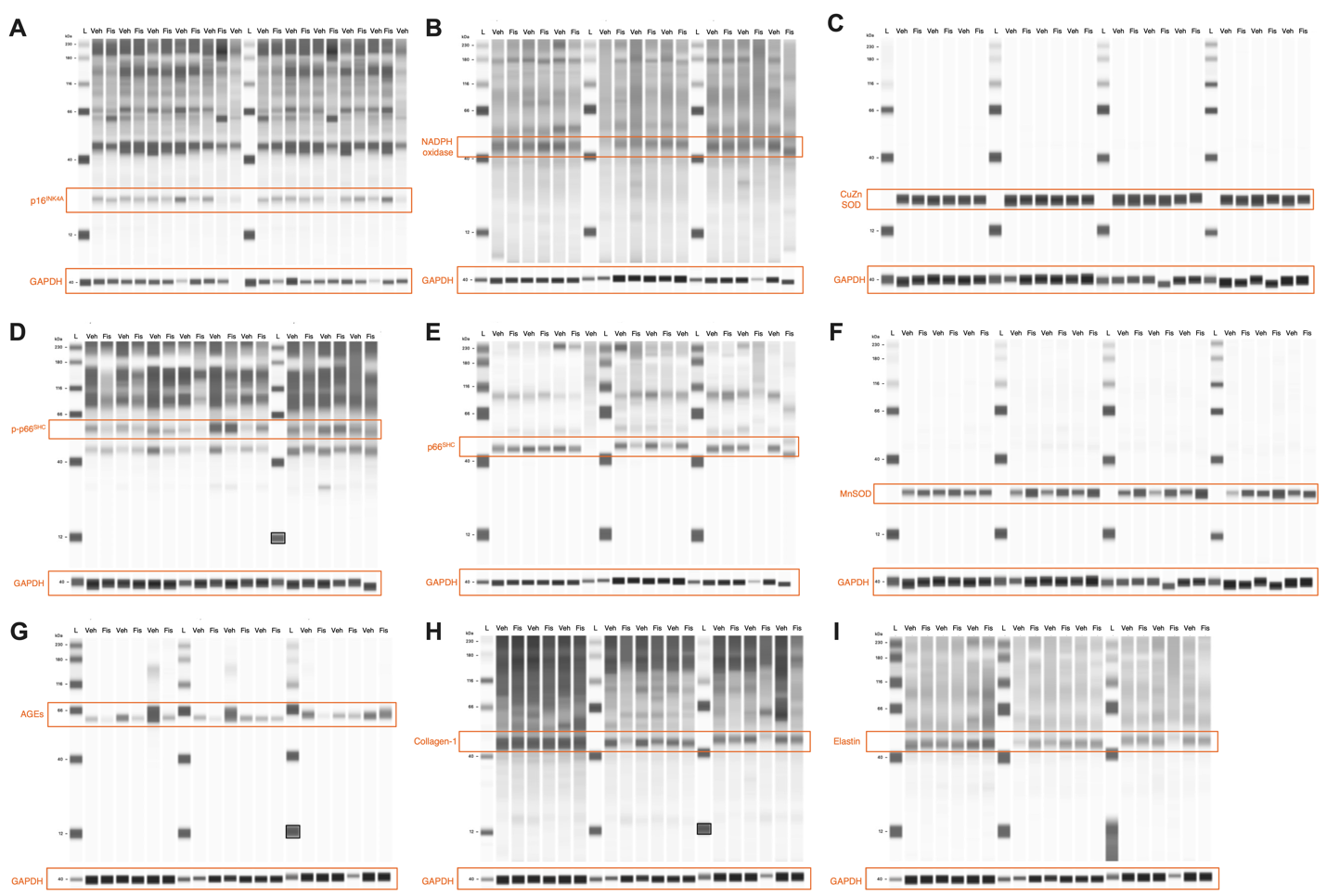


**
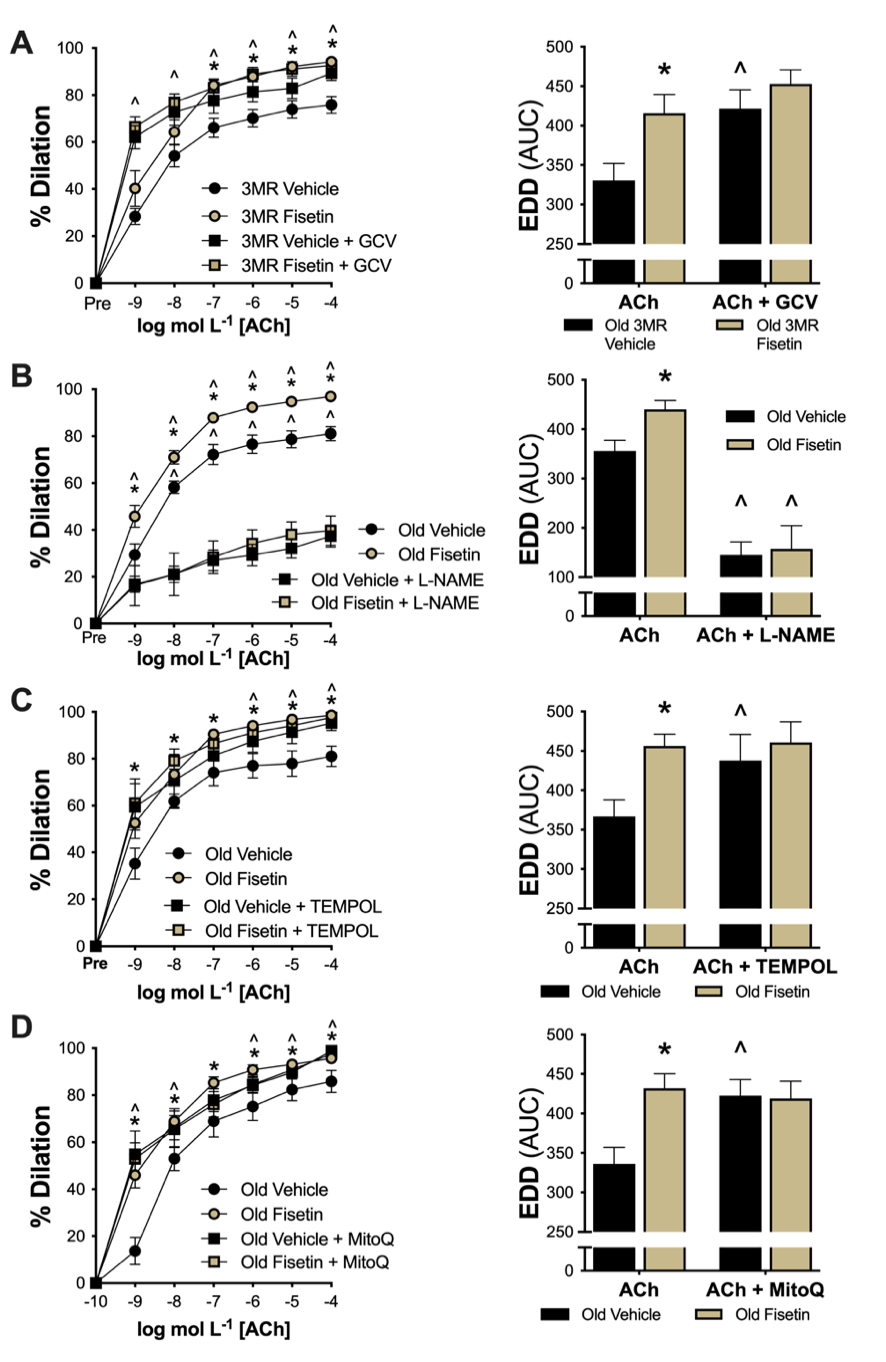
**

**Figure S3.** Endothelium-dependent dilation (EDD) curves and area under the curve (AUC) in isolated carotid arteries in response to acetylcholine (ACh; 1 X 10^-9^ to 1 X 10^-4^ M; n=12-18) and in the presence or absence of ganciclovir (GCV; 5μm; 180min pre-incubation; n=9-11) **(A)**, L-NAME (0.1mM; 30min pre-incubation; n=15) **(B)**, TEMPOL (1mM, 60min pre-incubation; n=7-8) **(C)**, and MitoQ (1µM; 60min pre-incubation; n=5-7) **(D)**. Values represent mean ± SEM. **P*<0.05 old vehicle vs. old fisetin; ^*P*<0.05 vs. ACh alone.

**Figure S4.** Aortic protein abundance to total p66^SHC^ with representative virtual blot bands. n=8-10/group. Values represent mean ± SEM.

**SUPPLEMNTAL REFERENCES**

Brunt, V. E., Gioscia-Ryan, R. A., Richey, J. J., Zigler, M. C., Cuevas, L. M., Gonzalez, A., . . . Seals, D. R. (2019). Suppression of the gut microbiome ameliorates age-related arterial dysfunction and oxidative stress in mice. *J Physiol, 597*(9), 2361-2378. doi:10.1113/JP277336

Casso, A. G., VanDongen, N. S., Gioscia-Ryan, R. A., Clayton, Z. S., Greenberg, N. T., Ziemba, B. P., . . . Brunt, V. E. (2022). Initiation of 3,3-dimethyl-1-butanol at midlife prevents endothelial dysfunction and attenuates in vivo aortic stiffening with ageing in mice. *J Physiol, 600*(21), 4633-4651. doi:10.1113/JP283581

Chang, J., Wang, Y., Shao, L., Laberge, R. M., Demaria, M., Campisi, J., . . . Zhou, D. (2016). Clearance of senescent cells by ABT263 rejuvenates aged hematopoietic stem cells in mice. *Nat Med, 22*(1), 78-83. doi:10.1038/nm.4010

Clayton, Z. S., Brunt, V. E., Hutton, D. A., Casso, A. G., Ziemba, B. P., Melov, S., . . . Seals, D. R. (2021). Tumor Necrosis Factor Alpha-Mediated Inflammation and Remodeling of the Extracellular Matrix Underlies Aortic Stiffening Induced by the Common Chemotherapeutic Agent Doxorubicin. *Hypertension, 77*(5), 1581-1590. doi:10.1161/HYPERTENSIONAHA.120.16759

Clayton, Z. S., Hutton, D. A., Brunt, V. E., VanDongen, N. S., Ziemba, B. P., Casso, A. G., . . . Seals, D. R. (2021). Apigenin restores endothelial function by ameliorating oxidative stress, reverses aortic stiffening, and mitigates vascular inflammation with aging. *Am J Physiol Heart Circ Physiol, 321*(1), H185-H196. doi:10.1152/ajpheart.00118.2021

Demaria, M., O'Leary, M. N., Chang, J., Shao, L., Liu, S., Alimirah, F., . . . Campisi, J. (2017). Cellular Senescence Promotes Adverse Effects of Chemotherapy and Cancer Relapse. *Cancer Discov, 7*(2), 165-176. doi:10.1158/2159-8290.CD-16-0241

Demaria, M., Ohtani, N., Youssef, S. A., Rodier, F., Toussaint, W., Mitchell, J. R., . . . Campisi, J. (2014). An essential role for senescent cells in optimal wound healing through secretion of PDGF-AA. *Dev Cell, 31*(6), 722-733. doi:10.1016/j.devcel.2014.11.012

Jo, J. H., Jo, J. J., Lee, J. M., & Lee, S. (2016). Identification of absolute conversion to geraldol from fisetin and pharmacokinetics in mouse. *J Chromatogr B Analyt Technol Biomed Life Sci, 1038*, 95-100. doi:10.1016/j.jchromb.2016.10.034

Kurz, D. J., Decary, S., Hong, Y., & Erusalimsky, J. D. (2000). Senescence-associated (beta)-galactosidase reflects an increase in lysosomal mass during replicative ageing of human endothelial cells. *J Cell Sci, 113 ( Pt 20)*, 3613-3622. doi:10.1242/jcs.113.20.3613

Lesniewski, L. A., Seals, D. R., Walker, A. E., Henson, G. D., Blimline, M. W., Trott, D. W., . . . Donato, A. J. (2017). Dietary rapamycin supplementation reverses age-related vascular dysfunction and oxidative stress, while modulating nutrient-sensing, cell cycle, and senescence pathways. *Aging Cell, 16*(1), 17-26. doi:10.1111/acel.12524

Schindelin, J., Arganda-Carreras, I., Frise, E., Kaynig, V., Longair, M., Pietzsch, T., . . . Cardona, A. (2012). Fiji: an open-source platform for biological-image analysis. *Nat Methods, 9*(7), 676-682. doi:10.1038/nmeth.2019

Scott, G. K., Yau, C., Becker, B. C., Khateeb, S., Mahoney, S., Jensen, M. B., . . . Benz, C. C. (2019). Targeting Mitochondrial Proline Dehydrogenase with a Suicide Inhibitor to Exploit Synthetic Lethal Interactions with p53 Upregulation and Glutaminase Inhibition. *Mol Cancer Ther, 18*(8), 1374-1385. doi:10.1158/1535-7163.MCT-18-1323
